# A benchmark for microRNA quantification algorithms using the OpenArray platform

**DOI:** 10.1186/s12859-016-0987-8

**Published:** 2016-03-22

**Authors:** Matthew N. McCall, Alexander S. Baras, Alexander Crits-Christoph, Roxann Ingersoll, Melissa A. McAlexander, Kenneth W. Witwer, Marc K. Halushka

**Affiliations:** Department of Biostatistics and Computational Biology, University of Rochester, 265 Crittenden Blvd, Rochester, 14642 NY USA; Department of Pathology, The Johns Hopkins University, 720 Rutland Ave, Baltimore, 21205 MD USA; Biology Department, The Johns Hopkins University, 3400 N Charles St, Baltimore, 21218 MD USA; Genetic Resources Core Facility, The Johns Hopkins University, 600 N Wolfe St, Baltimore, 21287 MD USA; Department of Molecular and Comparative Pathobiology, The Johns Hopkins University, 733 N Broadway, Baltimore, 21205 MD USA; Department of Neurology, The Johns Hopkins University, 855 N Wolfe St, Baltimore, 21205 MD USA

**Keywords:** microRNA, qPCR, Expression

## Abstract

**Background:**

Several techniques have been tailored to the quantification of microRNA expression, including hybridization arrays, quantitative PCR (qPCR), and high-throughput sequencing. Each of these has certain strengths and limitations depending both on the technology itself and the algorithm used to convert raw data into expression estimates. Reliable quantification of microRNA expression is challenging in part due to the relatively low abundance and short length of the miRNAs. While substantial research has been devoted to the development of methods to quantify mRNA expression, relatively little effort has been spent on microRNA expression.

**Results:**

In this work, we focus on the Life Technologies TaqMan OpenArray^Ⓡ^ system, a qPCR-based platform to measure microRNA expression. Several algorithms currently exist to estimate expression from the raw amplification data produced by qPCR-based technologies. To assess and compare the performance of these methods, we performed a set of dilution/mixture experiments to create a benchmark data set. We also developed a suite of statistical assessments that evaluate many different aspects of performance: accuracy, precision, titration response, number of complete features, limit of detection, and data quality. The benchmark data and software are freely available via two R/Bioconductor packages, **miRcomp** and **miRcompData**. Finally, we demonstrate use of our software by comparing two widely used algorithms and providing assessments for four other algorithms.

**Conclusions:**

Benchmark data sets and software are crucial tools for the assessment and comparison of competing algorithms. We believe that the **miRcomp** and **miRcompData** packages will facilitate the development of new methodology for microRNA expression estimation.

**Electronic supplementary material:**

The online version of this article (doi:10.1186/s12859-016-0987-8) contains supplementary material, which is available to authorized users.

## Background

MicroRNAs (miRNAs) are a class of small (18–24 nucleotide) regulatory RNAs. They are essential regulators that act as translational repressors throughout many eukaryotic species [1]. Several thousand miRNAs have been described in humans and other species, although in practicality only 350–400 are present at robust levels in mature cells and tissues [2]. MiRNAs are known to alter their expression levels in disease, malignancy, and cell stress [3] and exhibit tissue and cell-type specific patterns of expression [4, 5].

Many expression platforms, originally designed to quantify mRNA expression, have been adapted to globally assay miRNA expression including hybridization arrays, quantitative PCR (qPCR), and sequencing [6]. However, each of these approaches must overcome several challenges specific to miRNAs: short sequence length, low abundance of target molecules, and sequence homology between miRNAs. Comparative performance assessments are crucial to understanding the strengths and limitations of each approach to miRNA quantification. A group of investigators recently systematically evaluated 12 available miRNA platforms across 20 standardized control samples [7]. This study, called *miRQC*, established metrics to assay reproducibility, sensitivity, accuracy, specificity and concordance across the different methods. Although a single platform was not found to be uniformly superior, there was substantial variability in performance across assessments. For each of the platforms, performance depends on both the instrument and the algorithm used to convert raw measurements into expression estimates. For example, one platform assessed in the miRQC study was RNA-seq performed on an Illumina GAIIx instrument. The sample prep used the TruSeq Small RNA Prep Kit and results were aligned to the hg19 reference sequence allowing one mismatch, without further delineation of the alignment method [7]. A previous performance evaluation of miRNA expression arrays noted a strong dependency between technology and signal processing methodology [8]. More recently, we have demonstrated that different miRNA RNA-seq alignment algorithms produce different alignments, impacting the quality of the data [9]. We surmise that many miRNA expression platforms are not yet optimized to yield consistent and maximally accurate data.

Another platform evaluated in the miRQC study was the Life Technologies TaqMan OpenArray^Ⓡ^ system. This is a qPCR-based miRNA array platform that currently has coverage for 754 human miRNAs across two sets of primer pools. While qPCR is considered the gold standard for low-throughput measurement of gene expression, microarray- and sequencing-based platforms are preferable for most high-throughput applications. Given the relative small number of common miRNAs, it is possible to use a qPCR-based platform to measure the expression of all abundant miRNAs in many tissues and cells.

The primary advantage of qPCR-based technologies is the ability to simultaneously amplify and quantify a target transcript over sequential PCR cycles. The greater the initial amount of the target transcript present in a sample, the more rapidly the target will reach a threshold at which it can be detected by flourescence (e.g. from amplicon-associated intercalating dyes or freed, unquenched hydrolysis probes). As such, the raw data produced by qPCR-based technologies are fluorescence signal intensities captured at the end of each amplification cycle (typically 1–40). Analysis of these data typically begins by assigning a *threshold cycle* number to each amplification. These threshold cycles can then be used to estimate target abundance, either relative or in reference to values for a standard curve. For example, Life Technologies provides the ExpressionSuite software package, which uses the shape of the amplification curve to estimate a relative threshold cycle and corresponding expression estimate [10]. While a substantial number of software tools have been developed to estimate gene expression from raw amplification data [11–13], these focused on mRNA rather than miRNA targets. Whether these methods perform similarly when estimating miRNA expression is an area of ongoing research.

The software presented in this manuscript provides tools to assess and compare the performance of methods to transform raw amplification data into expression estimates and determine optimal quality thresholds. While the miRQC study focused on comparing many different platforms, here we focus on a single platform but provide a much larger and more diverse data set for evaluation. We believe that the availability of these data and corresponding software will greatly accelerate the development of improved methodology for the OpenArray^Ⓡ^ miRNA platform. Furthermore, seamless integration with the R/Bioconductor [14, 15] suite of analysis packages will enhance the value of OpenArray^Ⓡ^ miRNA data. Therefore, we developed **miRcomp**, an R package to assess and compare microRNA expression estimation methods using a benchmark data set.

## Methods

### Experimental design

#### Selection of tissues

Two separate RNA pools were prepared by blending two tissues each: (1) kidney and placenta and (2) skeletal muscle and brain (frontal cortex). These sources of RNA were chosen based on our prior analysis of Agilent V3 miRNA array data that suggested this collection of tissues would capture a large number of microRNAs, including several unique to each sample, such as miR-133a for skeletal muscle and the chromosome 19 miRNA cluster for placenta [2].

The surgical pathology archives of the Department of Pathology at Johns Hopkins Hospital were used to obtain formalin fixed paraffin-embedded (FFPE) tissues from four distinct tissue sources. All tissues were verified as normal by review of tissue histology on an adjacent hematoxylin and eosin stained slide. These anonymized human samples were used based on an exemption from the Institutional Review Board of Johns Hopkins Hospital.

#### RNA extraction

We extracted RNA from FFPE sections of kidney, placenta, skeletal muscle, and brain using the AllPrep DNA/RNA FFPE protocol (Qiagen). Xylene was chosen for deparaffinization. Extra xylene and ethanol washes were performed, and DNase digestion was done on-column.

#### RNA quality control

Concentration of eluted RNA was assessed by NanoDrop. Due to the low quality of longer RNA molecules extracted from FFPE tissues, including the ribosomal RNAs, the presence of several ubiquitous and tissue-enriched small RNAs or miRNAs was confirmed by stem-loop reverse transcription quantitative PCR using 10 ng RNA per reaction. For example, miR-1 and miR-133a were enriched in skeletal muscle, miR-516b was enriched in placenta, and miR-200b was enriched in kidney (Additional file 7: Figure S1). RNA was stored at −80C.

#### Reverse transcription and pre-amplification

**Fig. 1:**
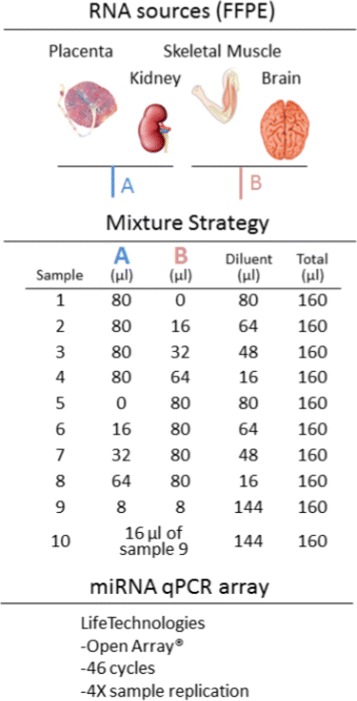
Two RNA pools were formed by blending two tissues each: (A) kidney and placenta and (B) skeletal muscle and brain. These pools were combined in different mixtures and dilutions to form the 10 sample types shown here. Gene expression in each sample type was assessed in quadruplicate (for a total of 40 samples) using the Life Technologies OpenArray^Ⓡ^ platform modified to perform 46 amplification cycles

The kidney/placenta (KP) and skeletal muscle/brain (MB) mixtures were made by combining equal masses of kidney and placenta or skeletal muscle and frontal cortex RNA, respectively, and diluting to an equal concentration of 3.3 ng/ul. 10 ng of RNA was used as the input for reverse transcription using the A and B primer pools, following the Life Technologies OpenArray^Ⓡ^ protocol modification for low-concentration and FFPE RNA. Separate reverse transcription and pre-amplification reactions were performed for the Life Technologies MegaPlex Pools A and B primer pools, which reverse transcribe and pre-amplify specific microRNAs. Following pre-amplification, 30 ul from the A and B reactions for both KP and MB were mixed with 570 ul of 0.1x TE. Further dilutions and combinations of the KP and MB mixtures were then prepared. To keep the non-nucelic acid components equal after mixing KP and MB, we added a diluent C mix as needed (Fig. 1). The diluent C included the same proportions of RT buffer and Pre-Amp mix components as in the Life Technologies protocol-specified dilution of nucleic acid-containing post-pre-amp mixture. The final concentrations were 50, 40, 20, 10 and 5 and 0.5 % for each sample (Fig. 1). The sample numbers (1–10 in Fig. 1) are used throughout the manuscript to refer to specific mixture/dilution sample types.

#### Life technologies openArray^Ⓡ^ assay

Standard Human TaqMan^Ⓡ^ OpenArray^Ⓡ^ Human MicroRNA Panel, QuantStudio ^*TM*^ 12K flex chips (part number 4470187) and other necessary reagents were provided by Life Technologies for this experiment. This panel contains 754 human miRNA sequences from miRBase v14 which have all been previously functionally validated with miRNA artificial templates. For conversion of notation from miRBase v14 style to current miRNA style, the webtool miRiadne can be used [16]. The specially prepared post-pre-amp dilution mixtures were added to the sample plates and then loaded onto the chips using the Accufill robot following the standard protocols (Life Technologies part number 4461306 Rev. B). A modified MicroRNA.edt file, provided by Life Technologies, was used to extend the cycles from the standard 40 to 46 cycles. This was done to make sure all amplifications went to completion, as the authors noted that some microRNA amplicons had not reached their maximal intensity at 40 cycles, causing a slight left shift to lower Crt values in prior experiments. The additional cycles do not increase the detection limit of the system. Three samples on one chip (the first replicate from sample types 1, 3, and 9) were run using the standard MicroRNA.edt file provided with the instrument, due to human error. This did not have a noticeable effect on the expression estimates from any of the algorithms. Additional information on the TaqMan^Ⓡ^ OpenArray^Ⓡ^ MicroRNA Panels can be found in the technical manual (Additional file 6).

### Expression estimation algorithms

There are a wide variety of algorithms available to estimate expression from qPCR amplification curves. To facilitate comparisons between these algorithms, we have applied many of these algorithms to our benchmark data set. The resulting expression estimates and quality scores are available as data objects in the **miRcomp** package.

Specifically, we provide expression estimates from the following methods:

- LifeTech ExpressionSuite
- 4 parameter sigmoidal model (b4)
- 5 parameter sigmoidal model (b5)
- 4 parameter log sigmoidal model (l4)
- 5 parameter log sigmoidal model (l5)
- Linear exponential model (linexp)

Additionally, the raw amplification data are available in the **miRcompData** package allowing researchers to easily generate expression estimates using other current or future algorithms.

### Statistical assessments

The primary goal of the mixture/dilution experiment described above is to provide a benchmark data set with which to assess the performance of methods that estimate miRNA expression from qPCR amplification curves. Specifically, we propose assessments of accuracy, precision, data quality, titration response, limit of detection, and number of complete features. Each of these is described in detail below. To avoid any confusion due to naming conventions (expression estimates from amplification curves have been called Ct values, Crt values, and Cq values to name a few), we refer to the reported values as *expression estimates* or simply *expression*.

#### Quality scores

When estimating expression from amplification data, it is crucial for methods to provide both an expression estimate and a corresponding quality score. These quality scores are often used to filter, flag, or down-weight poor quality expression estimates in subsequent analyses. The *qualityAssessment* function in the **miRcomp** package allows one to examine the relationship between quality scores and expression estimates, the distribution of quality scores across samples, and the relationship between quality scores from two different methods.

#### Expression comparison

When comparing two methods, a natural starting point is to compare the expression estimates produced by each method. By examining the features and samples for which expression estimates differ substantially, one can better understand the strengths and limitations of each method. The *expressionComp* function in the **miRcomp** package allows one to examine the relationship between expression estimates produced by two different methods. Feature/sample combinations for which the expression estimates differ by more than a given threshold are flagged for further investigation.

#### Complete features

A measure of the amount of readily usable data produced by a method is the number of complete features (here miRNAs). Complete features are defined as detected (non-NA expression estimate) and of good quality (above a given threshold) across all samples in a given experiment. The *completeFeatures* function allows one to assess a single method or compare two methods.

#### Limit of detection

The limit of detection is an estimate of the smallest signal that can be reliably measured. We propose assessing the limit of detection in two ways: (1) examining the distribution of average observed expression stratified by the proportion of values within a set of replicates that are good quality, and (2) comparing the average observed vs expected expression in the two low input sample types (9 & 10). The expected expression for both low input sample types (9 &10) can be calculated based on the pure sample types (1 & 5) or, in the case of the 0.01/0.01 dilution (sample type 10), it can be calculated based on the expression in the 0.1/0.1 dilution (sample type 9). Visual representations of these comparisons are produced by the *limitOfDetection* function.

The *limitOfDetection* function also reports several potential limits of detection based on each of the following comparisons:

1. Average observed expression in the 0.1/0.1 dilution samples (sample type 9) vs expected expression based on the pure samples (sample types 1 & 5).
2. Average observed expression in the 0.01/0.01 dilution samples (sample type 10) vs expected expression based on the pure samples (sample types 1 & 5).
3. Average observed expression in the 0.01/0.01 dilution samples (sample type 10) vs expected expression based on the 0.1/0.1 dilution samples (sample type 9).

For each of these comparisons, we calculate the difference between the observed and expected expression estimates. To assess the limit of detection, we compute the expression threshold such that the median difference (between observed and expected) of all features exceeding that threshold is equal to a predetermined tolerance. The *limitOfDetection* returns these potential limits of detection for each comparison and three tolerances (0.5, 0.75, and 1.00).

#### Titration response

The titration response is defined as the ability of a method to produce monotone increasing expression estimates in response to increasing amounts of input RNA. We consider sample types 2–4 and 6–8 as two separate titration series. In each of these series, one mixture component is held constant at 80 *μl* and the other is doubled twice from 16 *μl* to 32 *μl* to 64 *μl*. Because this response will depend heavily on the underlying expression of a given feature in each mixture component, the titration response is stratified by the difference in expression between the component being titrated and the component being held constant. For example, in the sample type 2–4 titration series, mixture component A is held constant and mixture component B is titrated. To assess the difference in expression between mixture components A and B, we use the expression estimates in the pure sample types: sample type 1 (pure A) and sample type 5 (pure B).

#### Accuracy

To assess accuracy, we calculate the signal detect slope, defined as the slope of the regression line of observed expression on expected expression, for the two titration series (sample types 2–4 &6–8). The ideal signal detect slope is one, representing agreement between observed and expected expression. The signal detect slopes are stratified by pure sample expression. A signal detect slope captures the average relationship between observed and expected expression; however, some features may perform well on average but be highly variable. In the plots produced, features are displayed in grey if the signal detect slope is not statistically significantly different from zero (*p*-value <0.05). As such, a grey point corresponding to a signal detect slope well above zero represents a particularly noisy (large residual variance) response.

#### Precision

To assess precision, we calculate both the within-replicate standard deviation and coefficient of variation (the within-replicate standard deviation divided by the within-replicate mean). Both statistics are calculated for each set of replicates (unique feature/sample type combinations) that are of acceptable quality. For both summaries, the values are stratified by the average observed expression.

### Software

Software implementing the assessments described in this manuscript was written in the open-source statistical language R (v3.2.1) [14]. The R software package, **miRcomp**, and the R data package, **miRcompData**, are available as part of the Bioconductor project [15] (v3.2 and later), a collaborative effort to develop software for computational biology and bioinformatics. In addition to the primary functionality described above, the **miRcomp** package contains many additional options for customizable use of these assessment functions. These are described in the **miRcomp** package vignette (included here as Additional file 1).

## Results

In the following, we compare two methods to generate expression estimates and quality scores from raw miRNA qPCR amplification data. The first method is an algorithm developed by Life Technologies and implemented in the ExpressionSuite software package. This software package produces estimates of expression (called Crt values) and a measure of quality (called the AmpScore). The second method is a four-parameter log-sigmoid curve-fitting algorithm [17] implemented as the default method in the **qpcR** R package [18] and referred to in this manuscript as simply *qpcR*. This open-source R package produces expression estimates by fitting a four parameter log sigmoidal curve to the amplification data and computing the point at which the second derivative of this curve is maximized (cpD2 method) [19] and a measure of quality (the *R*^2^ from the model fit).

Four additional algorithms (see Methods) were applied to the benchmark data set, and the resulting expression estimates and quality scores are available in the **miRcomp** R package. For clarity of presentation in this manuscript, we will focus on comparing two widely-used algorithms, the default algorithms from Life Technologies and the **qpcR** R package, in the following results.

### Quality assessment

**Fig. 2:**
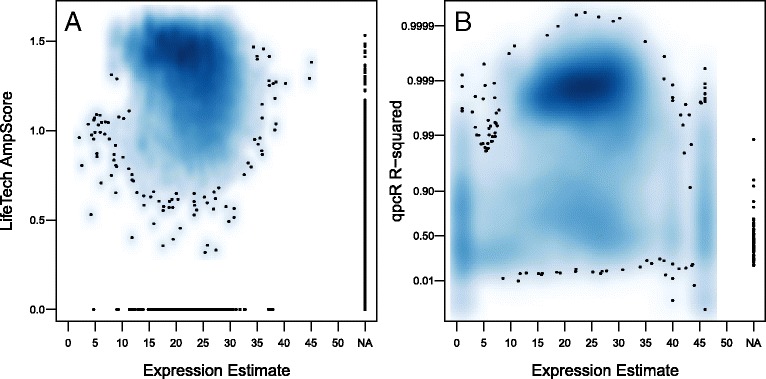
Relationship between quality and expression. For both data sets, a measure of data quality, (**a**) Life Technologies (LifeTech) AmpScore or (**b**) qpcR R-squared, is plotted against the corresponding expression estimates. The qpcR R-squared values are plotted on the complementary log-log scale to improve visibility in the region of interest. Each point represents a single expression measurement and corresponding quality measure for a unique miRNA/sample combination. Two-dimensional scatter-plot smoothing is used to avoid over-plotting and convey the distribution of points across the plotting region

Given the interdependence between the expression estimates and quality scores produced by a method, we begin by examining this relationship for each method (Fig. 2). As one might expect, quality scores decrease as the expression estimates increase (recall that for qPCR based technologies, a higher expression value corresponds to fewer copies of the target transcript). Another feature of note is that both methods occasionally fail to produce an expression estimate (denoted as NA in Fig. 2). However, while the qpcR method assigns all of these values fairly low quality (Fig. 2b), the Life Technologies method produces a substantial number of NA expression estimates with high quality scores (Fig. 2a).

**Fig. 3:**
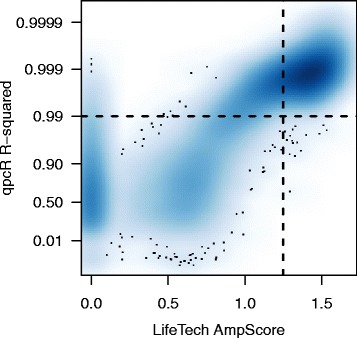
Comparison of quality scores. A direct comparison between the quality scores: Life Technologies (LifeTech) AmpScore and qpcR R-squared. The vertical dashed line represents the recommended AmpScore threshold of 1.25. The horizontal dashed line represents a potential R-squared threshold chosen by examination of this figure. Each point represents the quality values for a unique miRNA/sample combination. Two-dimensional scatter-plot smoothing is used to avoid over-plotting and convey the distribution of points across the plotting region

When comparing two methods, it is also interesting to examine the relationship between the quality scores produced by each method (Fig. 3). Examination of this figure highlights regions of consensus high quality (upper right) and consensus low quality (lower left) as well as regions of disagreement between the methods (upper left and lower right). While there are relatively few data points that are estimated with a high quality AmpScore and low quality *R*^2^, there are a substantial number of high quality *R*^2^ and low quality AmpScore data points.

Taken together, Figs. 2 & 3 suggest quality thresholds of AmpScore=1.25 and *R*^2^=0.99. While we will use these thresholds throughout the remainder of this manuscript, all functions in the **miRcomp** package allow the user to set their own quality thresholds. Furthermore, for many functions, one can compare results from a single method using two different quality thresholds to examine the effect of changing the quality threshold on each assessment. Lastly, when comparing two methods, we typically restrict the assessment to data considered to be good quality by both methods. This provides the most direct comparison between the expression estimates produced by the two methods; however, for many of these assessments, the **miRcomp** package allows one to perform these comparisons using each method’s own quality assessment independently.

### Expression comparison

**Fig. 4:**
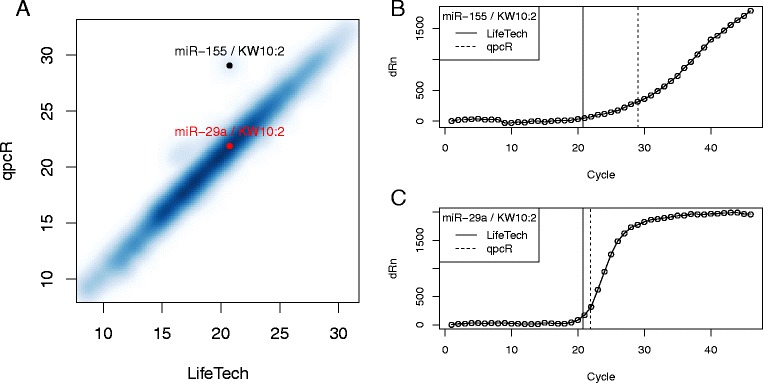
Comparison of expression estimates. **a** Expression estimates from Life Technologies (LifeTech) method and the default qpcR method are plotted against one another. The miRNA/sample combination in which the expression estimates differ most (miR-155; KW10:2) is represented by a black dot. An miRNA/sample combination in which the expression estimates roughly agree (miR-29a/KW10:2) is represented by a red dot. **b** & **c** The amplification curve data (*Δ*Rn vs cycle) for **b** miR-155 or **c** miR-29a in sample KW10:2 with expression estimates for each method denote by vertical lines. Note that the amplification curve is still increasing at cycle 46 for miR-155 whereas for miR-29a is has leveled off

When comparing two methods, it is also interesting to examine the relationship between the expression estimates produced by each method (Fig. 4a). Overall, the expression estimates produced by the Life Technologies and qpcR methods are quite similar; however, one feature/sample shows a substantial difference between methods (miR-155 in sample KW10:2). One can investigate this difference further by examining the raw amplification data provided in the **miRcompData** package. For comparison, we selected a feature from the same sample for which the two methods were approximate agreement (miR-29a in sample KW10:2). The amplification curve for miR-155 (Fig. 4b) shows the probable cause of the discrepency between expression estimates – the amplification curve is still increasing at the maximum observed cycle. In contrast, the amplification curve for miR-29a (Fig. 4c) has clearly reached a plateau before the maximum cycle. This results in expression estimates that are fairly similar across methods.

### Complete features

Given the difficulty in measuring the expression of many miRNAs, we examine the number of *complete features* (good quality across all 40 samples), *partial features* (good quality in at least one but not all samples), and *absent features* (poor quality in all 40 samples). Examining the number of features that fall into each of these categories for each method (Table 1) provides a straightforward assessment of the amount of easily usable data produced by each method. Of the 754 features in these data, there are 165 complete features using the Life Technologies method and 251 complete features using the qpcR method; however, this may simply be a result of the choice of quality threshold. Of note, there are 162 complete features in common between both methods and 103 features that are considered absent by both methods. The latter may represent miRNAs that are not expressed in any of the four tissues included in these data.

**Table 1:** Complete feature assessment

		qpcR method		
		Complete	Partial	Absent
LifeTech	Complete	162	3	0
Method	Partial	87	288	0
	Absent	2	109	103

Complete features are ones that are detected (non-NA expression estimate) and have good quality across all 40 samples. Partial features are those that are detected and good quality in at least one (but not all) of the 40 samples. Absent features are those that are not detected or of poor quality in all 40 samples. These values are used to compare the performance of the Life Technologies (LifeTech) and qpcR algorithms

### Limit of detection

**Fig. 5:**
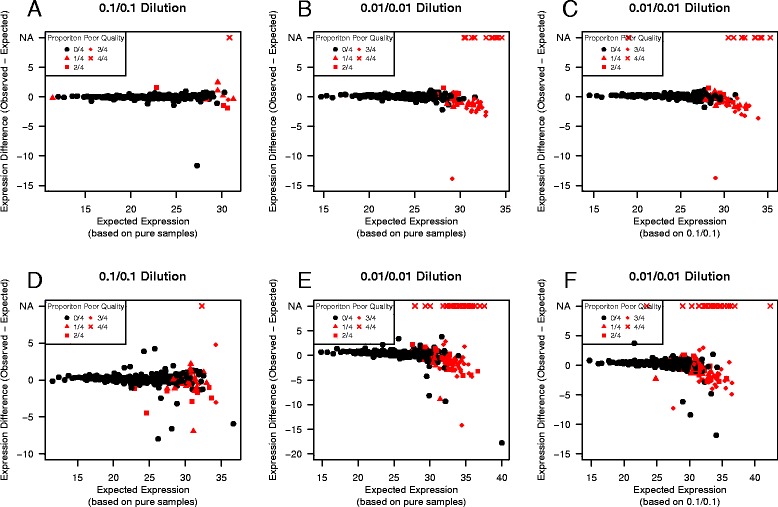
Limit of Detection MA-plots. To assess the limit of detection for a given method (Life Technologies **a**-**c**; qpcR **d**-**f**), we plot the difference between the observed and expected average expression vs the expected expression for each feature in the two low input sample types (either the 0.1/0.1 or 0.01/0.01 dilution samples). The expected expression is calculated using either the pure sample expression (**a**, **b**, **d**, & **e**) or the 0.1/0.1 dilution expression (**c** & **f**). Each point represents an miRNA in the sample type of interest. Features for which all four data points are of good quality are shown in black; other features are shown in red with the proportion of poor quality values for each feature denoted by plotting symbol. One can visually assess the limit of detect by the expected expression value (x-axis) at which the difference between observed and expected expression begins to differ substantially from zero and the proportion of poor quality values increases

Next, we examine the limit of detection for a given method. While this is related to the previous assessment of complete features, here the focus is on determining the minimum signal that can be reliably detected. The first assessment compares the difference between observed and expected expression vs the expected expression based on three different comparisons (Fig. 5). One can visually assess the limit of detection by identifying the expected expression value (x-axis) at which the expression difference (y-axis) begins to diverge from zero and the data are of increasingly poor quality. A quantitative version of this approach is described in the Methods Section. Potential limits of detection for each comparison and each tolerance are shown in Table 2. In general, the three comparisons produce similar limits of detection for a given tolerance.

**Table 2:** Potential limits of detection

	LifeTech method		
	0.1/0.1 vs pure	0.01/0.01 vs pure	0.01/0.01 vs 0.1/0.1
0.50	27.6	26.8	26.3
0.75	28.9	28.4	28.4
1.00	29.1	29.0	29.2
	qpcR Method		
	0.1/0.1 vs pure	0.01/0.01 vs pure	0.01/0.01 vs 0.1/0.1
0.50	26.9	25.8	25.3
0.75	29.2	28.3	28.6
1.00	30.1	29.3	29.9

Here we report three potential limits of detection based on the three assessments (0.1/0.1 vs pure, 0.01/0.01 vs pure, and 0.01/0.01 vs 0.1/0.1). Limits of detection are reported separately for the Life Technologies (LifeTech) and qpcR algorithms. Columns correspond to the different assessments, and rows correspond to the median difference between the observed and expected values. The values in the matrix are the expected expression values such that the median absolute difference for all larger expected expression values is approximately equal to the threshold for that row. For example, consider the Life Technologies method, if we focus on the 0.1/0.1 vs 0.01/0.01 comparison (column 3) and set a median average difference threshold of less than 1.00 (row 3), our estimate of the limit of detection is approximately 29.2

**Fig. 6:**
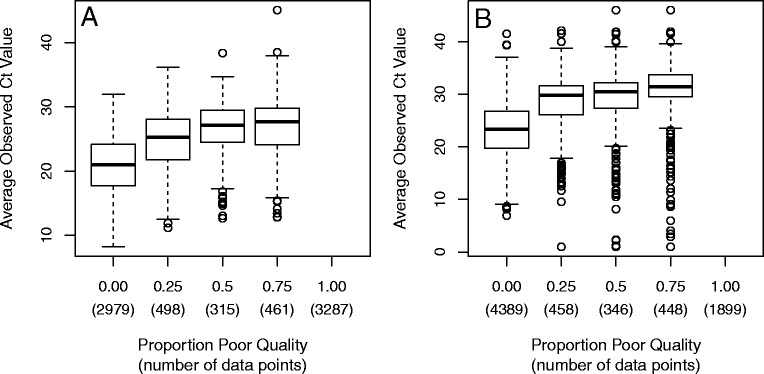
Limit of Detection Boxplots. To further examine the limit of detection, we examine boxplots of average observed expression stratified by the proportion of poor quality data points. Below each box, we also report the number of unique feature/sample type combinations each box contains. Results from the Life Technologies method are shown in panel **a**; results from the qpcR method are shown in panel **b**

An alternative approach to assess the limit of detection is to examine the distribution of average observed expression among replicates (unique feature/sample type combinations) stratified by the proportion of poor quality (Fig. 6). This allows one to easily see that higher expression values result in increasing amounts of poor quality data; however, for both methods, there is substantial overlap between the distributions.

### Titration response

**Fig. 7:**
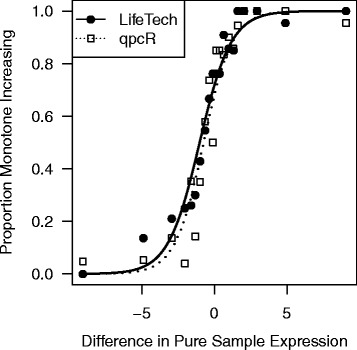
Titration Response. To examine the titration response, we plot the proportion of features that show monotone increasing expression as the amount of input RNA increases stratified by the difference in expression between the sample being titrated and the sample being held constant. Here we use samples 2–4 and 6–8 as two separate titration series. To assess the difference in expression between mixture components A and B, we use the expression estimates in the pure sample types: sample type 1 (pure A) and sample type 5 (pure B). Each point represents an average over the miRNAs falling within a given bin, where bins are defined based on the observed difference in pure sample expression (x-axis). The The Life Technologies (LifeTech) values are plotted with solid circles; the qpcR values with open squares

We now turn to assessments of the expression estimates themselves. For both methods, we calculated the proportion of features that show a titration response (see Methods for definition) for each titration series (Table 3). We also stratify these results by the difference in pure sample expression between the mixture component being titrated and the component being held constant (Fig. 7). As one might expect, if an miRNA is far less abundant in the component being titrated than in the component being held constant, it is difficult to detect a titration response for this miRNA. If an miRNA is far more abundant in the component being titrated than in the component being held constant, it is relatively easy to detect a titration response for this miRNA. While both methods perform fairly well when the features are expressed higher in the titrating component (x-axis value > 0), the Life Technologies method appears to perform slightly better for features expressed higher in the component being held constant; however, neither method performs particularly well for such features.

**Table 3:** Titration response

	LifeTech:A	qpcR:A	LifeTech:B	qpcR:B
Titration response	96	84	163	161
Non-response	108	120	41	43

This table displays the number of features that show a titration response (monotone increasing expression as the input RNA increases) for each method, Life Technologies (LifeTech) and qpcR, and each titration series (sample types 2–4 and 6–8). Here A and B refer to the mixture component being titrated: placenta & kidney (A) or skeletal muscle & brain (B)

### Accuracy

**Fig. 8:**
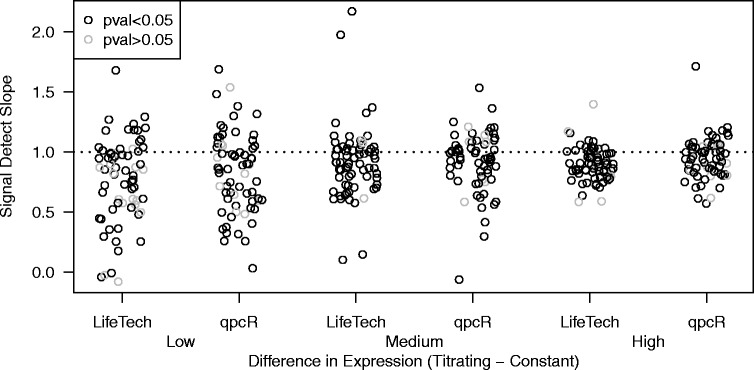
Accuracy Assessment. To assess accuracy, we calculate the signal detect slope: the slope of the regression line of observed expression on expected expression for both the Life Technologies (LifeTech) and qpcR algorithms. The ideal signal detect slope is one, representing agreement between observed and expected expression. The signal detect slopes are stratified by pure sample expression. Each point represents an miRNA. Points in the figures below are grey if the signal detect slope is not statistically signficantly different from zero. As such, a grey point corresponding to a signal detect slope well above zero represents a particularly noisy (large residual variance) response

Related to the titration response is the accuracy of the expression estimates. Rather than simply requiring monotone increasing expression estimates in response to increasing input RNA, here we are interested in the actual magnitude of the increase in expression. Specifically, to assess accuracy, we calculate the signal detect slope, defined as the slope of the regression line of observed expression on expected expression. We stratify the signal detect slopes into three equally sized bins to highlight the dependence on the underlying difference in expression noted in the previous subsection. Similar to the results seen in Fig. 7, accuracy is better for features with higher relative expression in the titrating component (Fig. 8). For each of the bins shown in Fig. 8, we can summarize the accuracy by computing robust measures of center and spread – median and median absolute deviation (MAD) – of the signal detect slopes (Table 4). The qpcR method appears to have slightly better accuracy, although this is not significant.

**Table 4:** Accuracy assessment

		LifeTech method	
	Low	Medium	High
Bin	(−0.0983,0.916]	(0.916,1.95]	(1.95,11.7]
Median	0.85	0.91	0.90
MAD	0.32	0.19	0.14
		qpcR method	
	Low	Medium	High
Bin	(−0.095,0.964]	(0.964,2.07]	(2.07,12]
Median	0.86	0.95	0.95
MAD	0.34	0.20	0.15

For each of the bins shown in Fig. 8, robust estimates of the center (median) and spread (MAD) of the signal detect slopes are reported for each method, Life Technologies (LifeTech) and qpcR

### Precision

**Fig. 9:**
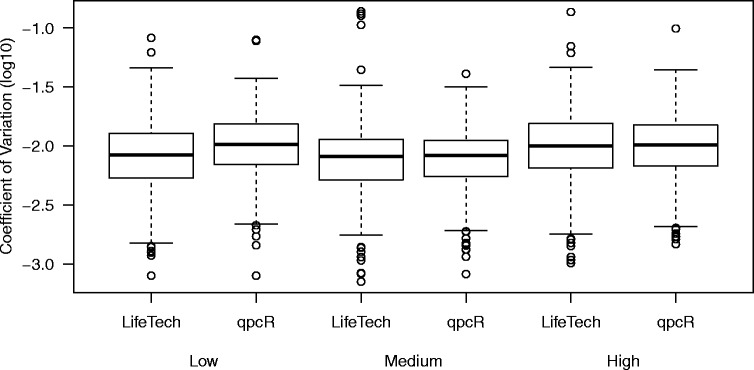
Precision Assessment. To assess precision, we calculate the within-replicate coefficient of variation, calculated as the within-replicate standard deviation divided by the within-replicate mean, for both the Life Technologies (LifeTech) and qpcR algorithms. These are calculated for each set of replicates (unique feature/sample type combination) that are of good quality and stratified by the observed expression

Finally, we consider a measure of precision, the within-replicate coefficient of variation. Here, replicates are defined as each unique feature/sample type combination consisting of four data points each. The data are divided into three bins of equal size based on the average expression of each replicate (Fig. 9). Both methods appear to have comparable precision.

### Performance of other algorithms

To this point, we have focused on assessing two algorithms to demonstrate the functionality of the **miRcomp** R/Bioconductor package. However, we also applied four additional algorithms (see Methods) to this benchmark data set. The resulting expression estimates and quality scores are available in the **miRcomp** R package. Performance assessments comparing all six algorithms are shown in Additional file 7: Figures S2–S7.

The relationship between expression estimates and quality scores was fairly similar across methods with the 5 parameter log sigmodial method producing slightly better quality scores and the linear exponential method producing overall lower quality scores (Additional file 7: Figure S2). The limit of detection was roughly 30 for all six algorithms, although the limit of detection appears slightly lower for the Life Technologies and linear exponential methods (Additional file 7: Figures S3 & S4). The Life Technologies and 5 parameter log sigmodial methods performed somewhat better in terms of titration response, while the linear exponential method performed significantly worse (Additional file 7: Figure S5). The linear exponential and 5 parameter log sigmoid methods had the best accuracy on average, but they also had the most variation in accuracy (Additional file 7: Figure S6). All methods performed better when the target transcript was highly expressed. The Life Technologies method had consistently the best precision and the 5 parameter log sigmoidal method had consistently the worst precision (Additional file 7: Figure S7).

## Discussion

In the previous section, we focused on assessing two algorithms to demonstrate the functionality of the **miRcomp** R/Bioconductor package. Additionally, we evaluated the performance of four other algorithms. In summary, the 4-parameter and 5-parameter sigmodial and log-sigmodial algorithms performed similarly, with the 5-parameter log-sigmoidal achieving slightly better accuracy by sacrificing some precision. In contrast the Life Technologies method appears to have sacrificed a bit of accuracy to achieve better precision. The linear exponential algorithm performed significantly worse than the other five methods.

However, the primary purpose of this work is to facilitate the development of new expression estimation algorithms by allowing researchers to assess their own method(s). The benchmark data set, described in the Methods section and provided in the **miRcompData** R/Bioconductor package, provides a rich resource for novel methods development. One can use the assessment functions in the **miRcomp** package to examine the effect of higher or lower quality thresholds for a currently available algorithm. We anticipate that these packages will facilitate the comparison of competing algorithms and guide the selection of those most suitable for a specific experiment. Furthermore, we anticipate that the development of open-source software to estimate expression from the raw amplification data will lead to increased integration between expression estimation procedures and subsequent statistical analyses, often performed with R/Bioconductor software packages.

Software to estimate expression from amplification data are implemented across a wide variety of operating systems (e.g. Windows, Mac OS, and Unix/Linx) and programming languages (e.g. R, Python, Perl, and SAS) [20]. As there is currently no standard data structure for raw qPCR amplification data, we store the raw data in a simple table that can be easily exported for the **miRcompData** package and converted into formats required by other expression estimation software. While the assessments described in this manuscript are implemented in R, the assessment functions accept simple matrices as input, which should be easy to create from the output of any other software package.

The assessment strategy proposed in this manuscript is based on a mixture/dilution experimental design. An alternative strategy would have been to spike-in known amounts of several miRNAs. Assessing accuracy for genomic technologies presents a challenge because the correct outcome for a given measurement must be known a priori. Spike-in experiments represent a natural way to accomplish this by comparing the nominal spike-in concentrations with the observed expression estimates. This is crucial to assess the accuracy of absolute expression estimates. However, performance assessments based on spike-in data depend strongly on the chosen spike-in concentrations [21, 22]. One must select spike-ins that span the entire dynamic range of observed expression, are unexpressed in all biological samples being analyzed, and are measured by the technology under consideration.

In a mixture/dilution experiment, the focus is on relative rather than absolute expression. While the absolute expression of a given feature is unknown, the relative expression is known. Therefore, in the assessments proposed in this manuscript, we have focused on the ability of various methods to accurately measure relative expression. Additionally, mixture/dilution experiments are inherently more realistic with respect to the dynamic range of observed expression levels by using mixtures of two biologically distinct RNA pools at varying proportions. Such a mixture/dilution approach allows one to characterize performance of assays across the full spectrum of expression, which is necessary to assess the performance of any analytic strategy. While one is restricted to assessments of relative expression, this corresponds to the standard use of qPCR expression assays – to measure differential rather than absolute expression.

## Conclusions

In this manuscript, we present a benchmark data set and software to assess the performance of methods that estimate expression from raw data on the Life Technologies OpenArray^Ⓡ^ microRNA platform. The raw data and software packages, **miRcomp** and **miRcompData**, are open-source and freely available as part of the Bioconductor project. We believe that the data and assessment software will facilitate the develop of novel methods for quantification of microRNA expression from the Life Technologies OpenArray^Ⓡ^ platform. Using these packages, we assessed the performance of six expression estimation algorithms. The 5-parameter log-sigmodial algorithm had the best accuracy; the Life Technologies method had the best titration response and precision.

## Availability of data and materials

R scripts to reproduce all figures and supplementary figures are included as Additional files 2 and 3. The datasets supporting the conclusions of this article are available in the miRcomp and miRcompData R/Bioconductor packages, included within the article as Additional files 4 and 5 and available online: http://bioconductor.org/packages/miRcomp/http://bioconductor.org/packages/miRcompData/.

## Additional files

Additional file 1 miRcomp package vignette. The miRcomp vignette describes standard use of the package as well as additional options and functionality not presented in this manuscript. (HTML 2017 kb)

Additional file 2 R script to create figures. The R script used to generate Figures 2–9 presented in this manuscript. (R 5.11 KB)

Additional file 3 R script to create supplementary figures. The R script used to generate Supplementary Figures 2-7. (R 5.33 KB)

Additional file 4 miRcomp R package source. The source code for the miRcomp R package, also available at: http://bioconductor.org/packages/miRcomp/. (GZ 1802 kb)

Additional file 5 miRcompData R package source. The source code for the miRcompData R package, also available at: http://bioconductor.org/packages/miRcompData/. (GZ 8765 kb)

Additional file 6 TaqMan OpenArray MicroRNA Panels User Guide. The technical manual of the source code for the TaqMan OpenArray MicroRNA Panels, also available at: https://tools.thermofisher.com/content/sfs/manuals/cms_092509.pdf. (PDF 996 KB)

Additional file 7 Supplementary Figures. A PDF containing 7 Supplementary Figures referred to in this manuscript. (PDF 770 kb)
