# Supplementary material for: A benchmark for microRNA quantification algorithms using the OpenArray platform
Source: BMC Bioinformatics. 2016 Mar 22;17:138. doi: 10.1186/s12859-016-0987-8 (PMC4802579; doi:10.1186/s12859-016-0987-8)
Supplement: Additional file 1 — miRcomp package vignette. The miRcomp vignette describes standard use of the package as well as additional options and functionality not presented in this manuscript. (HTML 2017 kb) [file 12859_2016_987_MOESM1_ESM.html]

Assessment and comparison of miRNA expression estimation methods (miRcomp)


# Assessment and comparison of miRNA expression estimation methods (miRcomp)

#### *Matthew N. McCall*

#### *2016-02-21*

# Contents

- 1 Introduction
  - 1.1 Background
  - 1.2 Experimental Design
- 2 Example Assessment
  - 2.1 Data Sets
  - 2.2 Quality Assessment
  - 2.3 Complete Features
  - 2.4 Limit of Detection
  - 2.5 Titration Response
  - 2.6 Accuracy
  - 2.7 Precision
- 3 Assessing a New Method
- 4 Session Info

# 1 Introduction

In this vignette we demonstrate the functionality of the *miRcomp* package. This package uses data from a dilution / mixture experiment to assess methods that estimate microRNA expression from qPCR amplification curves. Specifically, this package provides assessments of accuracy, precision, data quality, titration response, limit of detection, and complete features. Each of these is described in the following sections. To avoid any confusion due to naming conventions (expression estimates from amplification curves have been called Ct values, Crt values, and Cq values to name a few), we refer to the reported values as *expression estimates* or simply *expression*.

## 1.1 Background

Life Technologies has designed a qPCR miRNA array panel to work within their TaqMan® OpenArray® system. The current version of the array has coverage for 754 human miRNAs and additional non-human miRNAs for normalization controls/spike-ins across two primer pools. For raw data analysis, LifeTechnologies uses a closed ExpressionSuite software package to analyze qPCR data. There are a number of previously developed open-source packages to analyze raw qPCR fluorescence data; however, unlike microarray and RNAseq data, raw qPCR data are typically analyzed using the manufacturer’s software. In fact, several open-source packages take expression values (e.g. Ct, Crt, or Cq values) as input, ignoring how these values were estimated. To assess the potential benefits of alternative expression measures, we developed *miRcomp*, a benchmark data set and R/Bioconductor package to facilitate the development of new algorithms to preprocess LifeTechnologies® OpenArray® miRNA data and to provide tools to integrate the data into other software packages from the Bioconductor tool set.

## 1.2 Experimental Design

Two separate RNA pools were prepared by blending two tissues each: (1) kidney and placenta and (2) skeletal muscle and brain (frontal cortex). These sources of RNA were chosen based on prior analyses suggesting that the majority of microRNAs are expressed in at least one of these tissues and that several microRNAs are unique to each pool (e.g. miR-133a for skeletal muscle and the chromosome 19 miRNA cluster for placenta). We extracted RNA from formaldehyde-fixed, paraffin-embedded (FFPE) sections using the AllPrep DNA/RNA FFPE protocol (Qiagen). Mixtures of RNA were made by combining equal masses of kidney and placenta or skeletal muscle and frontal cortex RNA, respectively, and diluting to equal concentration of 3.3 ng/ul. 10 ng of RNA was used as the input for reverse transcription using the ‘A’ and ‘B’ primer pools and following the Life Technologies Open Array protocol modification for low-concentration, FFPE RNA. Separate reverse transcription and pre-amplification reactions were performed for the Life Technologies MegaPlex Pools ‘A’ and ‘B’ primer pools. Following pre-amplification, 30 ul from the ‘A’ and ‘B’ reactions for both pools were mixed with 570 ul of 0.1x TE. Further dilutions and combinations of the pools were then prepared according to the following design:

Each of the 10 unique mixture / dilution sample types was performed four times.

# 2 Example Assessment

## 2.1 Data Sets

In the following, we will use two data sets included in the *miRcomp* package. The first was generated using the LifeTech ExpressionSuite software package. The second was generated using the default algorithm from the *qpcR* R package.

We first load the package and data.

```
## Load libraries
library('miRcomp')
```

```
data(lifetech)
data(qpcRdefault)
```

Each of the example data sets includes both an expression estimate (ct) and an assessment of the quality of each estimate (qc) for each of the 754 microRNAs and 40 samples. For the *lifetech* data set, the measure of quality is the AmpScore (a proprietary method of assessing the quality of an amplification curve). For the *qpcRdefault* data set, the measure of quality is the R2 value from the sigmoidal model fit to the amplification curve data.

```
str(lifetech)
```

```
## List of 2
##  $ ct: num [1:754, 1:40] 28.2 19.8 11.9 15.7 23.7 ...
##   ..- attr(*, "dimnames")=List of 2
##   .. ..$ : chr [1:754] "hsa-miR-409-5p_002331:A" "hsa-miR-424_000604:A" "hsa-miR-30b_000602:A" "hsa-miR-29a_002112:A" ...
##   .. ..$ : chr [1:40] "KW1:1" "KW1:2" "KW1:3" "KW1:4" ...
##  $ qc: num [1:754, 1:40] 1.29 1.33 1.42 1.45 1.17 ...
##   ..- attr(*, "dimnames")=List of 2
##   .. ..$ : chr [1:754] "hsa-miR-409-5p_002331:A" "hsa-miR-424_000604:A" "hsa-miR-30b_000602:A" "hsa-miR-29a_002112:A" ...
##   .. ..$ : chr [1:40] "KW1:1" "KW1:2" "KW1:3" "KW1:4" ...
```

```
str(qpcRdefault)
```

```
## List of 2
##  $ ct: num [1:754, 1:40] 29.8 21.1 12.6 16.6 25.2 ...
##   ..- attr(*, "dimnames")=List of 2
##   .. ..$ : chr [1:754] "hsa-miR-409-5p_002331:A" "hsa-miR-424_000604:A" "hsa-miR-30b_000602:A" "hsa-miR-29a_002112:A" ...
##   .. ..$ : chr [1:40] "KW1:1" "KW1:2" "KW1:3" "KW1:4" ...
##  $ qc: num [1:754, 1:40] 0.998 0.999 0.999 0.999 0.998 ...
##   ..- attr(*, "dimnames")=List of 2
##   .. ..$ : chr [1:754] "hsa-miR-409-5p_002331:A" "hsa-miR-424_000604:A" "hsa-miR-30b_000602:A" "hsa-miR-29a_002112:A" ...
##   .. ..$ : chr [1:40] "KW1:1" "KW1:2" "KW1:3" "KW1:4" ...
```

The colnames of the example data matrices correspond to the sample types shown in the design figure above. Each name starts with KW, followed by the sample type (1-10), then a colon and the replicate number (1-4).

```
colnames(lifetech$ct)
```

```
##  [1] "KW1:1"  "KW1:2"  "KW1:3"  "KW1:4"  "KW2:1"  "KW2:2"  "KW2:3" 
##  [8] "KW2:4"  "KW3:1"  "KW3:2"  "KW3:3"  "KW3:4"  "KW4:1"  "KW4:2" 
## [15] "KW4:3"  "KW4:4"  "KW5:1"  "KW5:2"  "KW5:3"  "KW5:4"  "KW6:1" 
## [22] "KW6:2"  "KW6:3"  "KW6:4"  "KW7:1"  "KW7:2"  "KW7:3"  "KW7:4" 
## [29] "KW8:1"  "KW8:2"  "KW8:3"  "KW8:4"  "KW9:1"  "KW9:2"  "KW9:3" 
## [36] "KW9:4"  "KW10:1" "KW10:2" "KW10:3" "KW10:4"
```

## 2.2 Quality Assessment

We begin by examing the quality scores provided by each of the example methods. The *qualityAssessment* function allows one to examine the relationship between quality scores and expression estimates (`plotType="scatter"`) or the distribution of quality scores across samples (`plotType="boxplot"`).

```
qualityAssessment(lifetech, plotType="scatter", label1="LifeTech AmpScore")
```

```
qualityAssessment(lifetech, plotType="boxplot", label1="LifeTech AmpScore")
```

In addition to assessing a single method, one has the option to compare two methods by passing an optional second data object to the function. For the scatter plot, this results in plotting the quality metrics against each other. For the boxplots, the results are simply presented in a single figure. One also has the option to apply the complementary log-log transformation to the quality metrics prior to plotting (e.g. `cloglog2=TRUE`). This is often useful for R2 quality metrics.

```
qualityAssessment(lifetech, object2=qpcRdefault, cloglog2=TRUE, plotType="scatter", label1="LifeTech AmpScore", label2="qpcR R-squared")
```

Finally, one can filter quality scores corresponding to NA expression estimates from the boxplots. This can be useful to focus on cases in which the expression estimates appear valid but may be of poor quality.

```
qualityAssessment(lifetech, plotType="boxplot", na.rm=TRUE, label1="LifeTech AmpScore")
```

## 2.3 Complete Features

Given the difficulty in measuring many microRNAs, we examine the number of complete features (here microRNAs). Complete features are ones that are detected (non-NA expression estimate) and good quality across all 40 samples. The *completeFeatures* function allows one to assess a single method or compare two methods.

```
completeFeatures(lifetech, qcThreshold1=1.25, label1="LifeTech")
```

```
##                                             LifeTech
## Complete miRNAs (all good quality & non-NA)      165
## Partial miRNAs (some good quality & non-NA)      375
## Absent miRNAs (no good quality & non-NA)         214
```

```
completeFeatures(lifetech, qcThreshold1=1.25, object2=qpcRdefault, qcThreshold2=0.99, label1="LifeTech", label2="qpcR")
```

```
##                   qpcR:Complete qpcR:Partial qpcR:Absent
## LifeTech:Complete           162            3           0
## LifeTech:Partial             87          288           0
## LifeTech:Absent               2          109         103
```

One can also use this function to compare two quality thresholds for the same expression estimates.

```
completeFeatures(lifetech, qcThreshold1=1.25, object2=lifetech, qcThreshold2=1.4, label1="LT 1.25", label2="LT 1.4")
```

```
##                  LT 1.4:Complete LT 1.4:Partial LT 1.4:Absent
## LT 1.25:Complete              48            117             0
## LT 1.25:Partial                0            255           120
## LT 1.25:Absent                 0              0           214
```

## 2.4 Limit of Detection

We also directly examine the limit of detection for a given method. While this is related to the previous assessment, here the focus is on determining the minimum signal that can be reliably detected. This is accomplished in three ways: examining the distribution of average observed expression stratfied by the proportion of values within a set of replicates that are good quality (`plotType="boxplot"`), plotting the average observed expression in the two low input sample types (9 & 10) vs the expected expression (`plotType="scatterplot"`), or plotting the difference between the average observed expression in the two low input sample types and the expected expression vs the expected expression (`plotType="MAplot"`). The expected expression for both low input sample types (9 & 10) can be calculated based on the pure sample types (1 & 5) or, in the case of the 0.01/0.01 dilution (sample type 10), it can be calculated based on the expression in the 0.1/0.1 dilution (sample type 9).

```
par(mar=c(6,6,2,2))
boxes <- limitOfDetection(lifetech, qcThreshold=1.25, plotType="boxplot")
```

```
str(boxes)
```

```
## List of 5
##  $ 0.00: num [1:2979] 19.5 11.9 15.9 16.4 26.4 ...
##  $ 0.25: num [1:498] 25.9 15.1 16.4 26.2 16.2 ...
##  $ 0.5 : num [1:315] 24.7 30.5 19.7 26.3 31.5 ...
##  $ 0.75: num [1:461] 28.2 24 31 28.3 34.4 ...
##  $ 1.00: logi [1:3287] NA NA NA NA NA NA ...
```

```
par(mfrow=c(1,3))
lods <- limitOfDetection(lifetech, qcThreshold=1.25, plotType="scatter")
```

```
par(mfrow=c(1,3))
lods <- limitOfDetection(lifetech, qcThreshold=1.25, plotType="MAplot")
```

```
print(round(lods,digits=2))
```

```
##      0.1/0.1 vs pure 0.01/0.01 vs pure 0.01/0.01 vs 0.1/0.1
## 0.50            27.6              26.8                 26.3
## 0.75            28.9              28.4                 28.4
## 1.00            29.1              29.0                 29.2
```

In all three cases, the function also returns additional information. For `plotType="boxplot"` the function returns the values in each box. For the other two plotTypes, the function returns several potential limits of detection. Specifically, it returns a matrix with three columns corresponding to the three figures and three rows corresponding to the median difference between the observed and expected values. The values in the matrix are the expected expression values such that the median absolute difference for all larger expected expression values is approximately equal to the threshold for that row. For example, in the output shown above, if we focus on the 0.1/0.1 vs 0.01/0.01 comparison (column 3) and set a median average difference threshold of less than 1.00 (row 3), our estimate of the limit of detection is approximately 29.2.

## 2.5 Titration Response

We now turn to assessments of the expression estimates themselves. Perhaps the most straight-forward assessment of a dilution experiment is the titration response, which features a consistent increase in expression with increasing amounts of input RNA. The *titrationResponse* function allows one to assess a single method or compare two methods. The output is a table displaying the number of features that show a titration response (monotone increasing expression as the input RNA increases). Here we use samples 2-4 and 6-8 as two separate titration series. The function also produces a figure showing the titration response stratified by the difference in expression between the sample being titrated and the sample being held constant. For example, in the sample type 2-4 titration series, mixture component A is held constant and mixture component B is titrated. To assess the difference in expression between mixture components A and B, we use the expression estimates in the pure sample types: sample type 1 (pure A) and sample type 5 (pure B).

```
titrationResponse(lifetech, qcThreshold1=1.25)
```

```
##            A   B
## Mono      98 164
## Non-Mono 109  43
```

```
titrationResponse(lifetech, qcThreshold1=1.25, object2=qpcRdefault, qcThreshold2=0.99, label1="LifeTech", label2="qpcR")
```

```
##          LifeTech:A qpcR:A LifeTech:B qpcR:B
## Mono             96     84        163    161
## Non-Mono        108    120         41     43
```

Note that the number features included in the assessment differs between the two tables shown above. This is due to the fact that when comparing two methods, a given feature must be of acceptable quality according to both methods (by default). However, one can remove this constraint by setting *commonFeatures=FALSE*.

```
titrationResponse(lifetech, qcThreshold1=1.25, object2=qpcRdefault, qcThreshold2=0.99, commonFeatures=FALSE, label1="LifeTech", label2="qpcR")
```

```
##          LifeTech:A qpcR:A LifeTech:B qpcR:B
## Mono             98    125        164    241
## Non-Mono        109    216         43    100
```

Finally, one can also compare two quality thresholds for the same expression estimates by passing the same object twice and setting two different quality thresholds. Clearly, *commonFeatures* must be set to FALSE when comparing two quality thresholds for the same expression estimates.

```
titrationResponse(lifetech, qcThreshold1=1.25, object2=lifetech, qcThreshold2=1.4, commonFeatures=FALSE, label1="AmpScore 1.25", label2="AmpScore 1.4")
```

```
##          AmpScore 1.25:A AmpScore 1.4:A AmpScore 1.25:B AmpScore 1.4:B
## Mono                  98             31             164             46
## Non-Mono             109             23              43              8
```

## 2.6 Accuracy

Related to the titration response is the accuracy of the expression estimates. Rather than simply requiring montone increasing expression estimates in response to increasing input RNA, here we are interested in the actual magnitude of the increase in expression. Specifically, to assess accuracy, we calculate the signal detect slope: the slope of the regression line of observed expression on expected expression. The ideal signal detect slope is one, representing agreement between observed and expected expression. Like many of the previous assessment functions, the *accuracy* function allows one to assess a single method or compare two methods. In both situations, the signal detect slopes are stratified by pure sample expression. Points in the figures below are grey if the signal detect slope is not statistically signficantly different from zero. As such, a grey point corresponding to a signal detect slope well above zero represents a particularly noisy (large residual variance) response.

```
accuracy(lifetech, qcThreshold1=1.25)
```

```
##        bin1              bin2           bin3         
## Bin    "(-0.0983,0.942]" "(0.942,1.97]" "(1.97,11.7]"
## Median "0.85"            "0.9"          "0.9"        
## MAD    "0.28"            "0.18"         "0.14"
```

```
accuracy(lifetech, qcThreshold1=1.25, object2=qpcRdefault, qcThreshold2=0.99, label1="LifeTech", label2="qpcR")
```

```
## $LifeTech
##        bin1              bin2           bin3         
## Bin    "(-0.0983,0.916]" "(0.916,1.95]" "(1.95,11.7]"
## Median "0.85"            "0.91"         "0.9"        
## MAD    "0.32"            "0.19"         "0.14"       
## 
## $qpcR
##        bin1             bin2           bin3       
## Bin    "(-0.095,0.964]" "(0.964,2.07]" "(2.07,12]"
## Median "0.86"           "0.95"         "0.95"     
## MAD    "0.34"           "0.2"          "0.15"
```

Similar to the titration response assessment, by default the accuracy function only considers features considered high quality based on both methods. However, this constraint can be removed by setting *commonFeatures=FALSE*.

```
accuracy(lifetech, qcThreshold1=1.25, object2=qpcRdefault, qcThreshold2=0.99, commonFeatures=FALSE, label1="LifeTech", label2="qpcR")
```

```
## $LifeTech
##        bin1              bin2           bin3         
## Bin    "(-0.0983,0.942]" "(0.942,1.97]" "(1.97,11.7]"
## Median "0.85"            "0.9"          "0.9"        
## MAD    "0.28"            "0.18"         "0.14"       
## 
## $qpcR
##        bin1            bin2          bin3         
## Bin    "(-0.095,1.08]" "(1.08,2.53]" "(2.53,17.8]"
## Median "0.83"          "0.93"        "0.94"       
## MAD    "0.36"          "0.24"        "0.16"
```

Finally, one can also compare two quality thresholds for the same expression estimates by passing the same object twice and setting two different quality thresholds. Clearly, *commonFeatures* must be set to FALSE when comparing two quality thresholds for the same expression estimates.

```
accuracy(lifetech, qcThreshold1=1.25, object2=lifetech, qcThreshold2=1.4, commonFeatures=FALSE, label1="AmpScore 1.25", label2="AmpScore 1.4")
```

```
## $`AmpScore 1.25`
##        bin1              bin2           bin3         
## Bin    "(-0.0983,0.942]" "(0.942,1.97]" "(1.97,11.7]"
## Median "0.85"            "0.9"          "0.9"        
## MAD    "0.28"            "0.18"         "0.14"       
## 
## $`AmpScore 1.4`
##        bin1             bin2          bin3         
## Bin    "(-0.0727,0.57]" "(0.57,1.61]" "(1.61,10.9]"
## Median "0.83"           "0.96"        "0.96"       
## MAD    "0.3"            "0.16"        "0.13"
```

## 2.7 Precision

Finally, we consider two measures of precision, the within-replicate standard deviation and coefficient of variation. The latter is calculated as the within-replicate standard deviation divided by the within-replicate mean. Both statistics are calculated for each set of replicates (unique feature / sample type combination) that are of good quality and stratified by the observed expression.

```
boxes <- precision(lifetech, qcThreshold1=1.25, statistic="sd")
```

```
str(boxes)
```

```
## List of 3
##  $ (8.58,17.4]: num [1:550] 0.1683 0.1148 0.1716 0.1346 0.0377 ...
##  $ (17.4,20.7]: num [1:550] 0.171 0.218 0.302 0.213 0.311 ...
##  $ (20.7,30.2]: num [1:550] 0.104 0.0674 0.4255 0.4014 0.4842 ...
```

```
boxes <- precision(lifetech, qcThreshold1=1.25, statistic="cv", bins=4)
```

```
str(boxes)
```

```
## List of 4
##  $ (8.58,16.6]: num [1:413] 0.01415 0.00981 0.01494 0.01229 0.00322 ...
##  $ (16.6,19]  : num [1:412] 0.00962 0.01285 0.01244 0.0161 0.01166 ...
##  $ (19,21.5]  : num [1:412] 0.00501 0.00323 0.02014 0.02117 0.01126 ...
##  $ (21.5,30.2]: num [1:413] 0.01813 0.01711 0.01961 0.00707 0.00962 ...
```

At times it can be beneficial to look at a scaled version of either the standard deviation or coefficient of variation. This can be easily accomplished by using `scale="log"` or `scale="log10"`.

```
boxes <- precision(lifetech, qcThreshold1=1.25, object2=qpcRdefault, qcThreshold2=0.99, scale="log", label1="LifeTech", label2="qpcR")
```

```
str(boxes)
```

```
## List of 6
##  $ LifeTech:(8.58,17.5]: num [1:540] -1.78 -2.16 -1.76 -2.01 -3.28 ...
##  $ qpcR:(8.97,18.3]    : num [1:540] -1.87 -2.24 -1.74 -1.67 -2.24 ...
##  $ LifeTech:(17.5,20.7]: num [1:540] -1.76 -1.53 -1.2 -1.55 -1.17 ...
##  $ qpcR:(18.3,21.8]    : num [1:540] -1.71 -1.55 -2.31 -1.78 -1.69 ...
##  $ LifeTech:(20.7,30.2]: num [1:540] -2.263 -2.697 -0.854 -0.913 -0.725 ...
##  $ qpcR:(21.8,31.4]    : num [1:540] -1.655 -2.209 -1.211 -1.344 -0.959 ...
```

```
boxes <- precision(lifetech, qcThreshold1=1.25, object2=qpcRdefault, qcThreshold2=0.99, statistic="cv", scale="log10", label1="LifeTech", label2="qpcR")
```

```
str(boxes)
```

```
## List of 6
##  $ LifeTech:(8.58,17.5]: num [1:540] -1.85 -2.01 -1.83 -1.91 -2.49 ...
##  $ qpcR:(8.97,18.3]    : num [1:540] -1.91 -2.06 -1.83 -1.78 -2.05 ...
##  $ LifeTech:(17.5,20.7]: num [1:540] -2.02 -1.91 -1.79 -1.93 -1.76 ...
##  $ qpcR:(18.3,21.8]    : num [1:540] -2.02 -1.94 -2.29 -2.04 -2.06 ...
##  $ LifeTech:(20.7,30.2]: num [1:540] -2.3 -2.49 -1.74 -1.77 -1.71 ...
##  $ qpcR:(21.8,31.4]    : num [1:540] -2.06 -2.3 -1.92 -1.97 -1.83 ...
```

Similar to the titration response assessment, by default the accuracy function only considers features considered high quality based on both methods. However, this constraint can be removed by setting *commonFeatures=FALSE*.

```
boxes <- precision(lifetech, qcThreshold1=1.25, object2=qpcRdefault, qcThreshold2=0.99, commonFeatures=FALSE, statistic="cv", scale="log10", label1="LifeTech", label2="qpcR")
```

```
str(boxes)
```

```
## List of 6
##  $ LifeTech:(8.58,17.4]: num [1:550] -1.85 -2.01 -1.83 -1.91 -2.49 ...
##  $ qpcR:(8.04,19]      : num [1:837] -1.91 -2.06 -1.83 -1.78 -2.05 ...
##  $ LifeTech:(17.4,20.7]: num [1:550] -2.02 -1.91 -1.79 -1.93 -1.76 ...
##  $ qpcR:(19,22.6]      : num [1:837] -1.82 -2.02 -1.9 -2.64 -1.78 ...
##  $ LifeTech:(20.7,30.2]: num [1:550] -2.3 -2.49 -1.74 -1.77 -1.71 ...
##  $ qpcR:(22.6,36]      : num [1:836] -1.93 -1.94 -1.97 -1.6 -1.63 ...
```

Finally, one can also compare two quality thresholds for the same expression estimates by passing the same object twice and setting two different quality thresholds. Clearly, *commonFeatures* must be set to FALSE when comparing two quality thresholds for the same expression estimates.

```
boxes <- precision(lifetech, qcThreshold1=1.25, object2=lifetech, qcThreshold2=1.4, commonFeatures=FALSE, statistic="cv", scale="log10", label1="AmpScore 1.25", label2="AmpScore 1.4")
```

```
str(boxes)
```

```
## List of 6
##  $ AmpScore 1.25:(8.58,17.4]: num [1:550] -1.85 -2.01 -1.83 -1.91 -2.49 ...
##  $ AmpScore 1.4:(9.49,17.2] : num [1:160] -1.96 -2.32 -2.08 -1.56 -1.99 ...
##  $ AmpScore 1.25:(17.4,20.7]: num [1:550] -2.02 -1.91 -1.79 -1.93 -1.76 ...
##  $ AmpScore 1.4:(17.2,19.9] : num [1:160] -2.02 -2.32 -2.09 -2.21 -2.22 ...
##  $ AmpScore 1.25:(20.7,30.2]: num [1:550] -2.3 -2.49 -1.74 -1.77 -1.71 ...
##  $ AmpScore 1.4:(19.9,28.4] : num [1:160] -2.15 -2.21 -2.52 -1.91 -2.17 ...
```

# 3 Assessing a New Method

So far we have focused on assessing two data sets that are available as part of the *miRcomp* package. However, miRcomp was designed to allow researchers to develop their own expression estimation algorithm and compare it to the algorithms available in the LifeTech Expression Suite and the *qpcR* R package. The novel benchmark data set described in the Experimental Design section and provided in the *miRcompData* package provides a rich resource for novel methods development. We anticipate that this package will facilitate the comparison of competing algorithms and guide the selection of those most suitable for a specific experiment. Furthermore, we predict that the development of open-source software to estimate expression from the raw amplification data will lead to increased intergration between expression estimation and subsequent statistical analyses, typically performed with currently available R/Bioconductor tools.

Whenever possible, we will include the expression estimates and quality metrics from new algorithms in the *miRcompData* package.

To load the raw amplification data:

```
library(miRcompData)
data(miRcompData)
```

The raw data are organized as follows:

```
str(miRcompData)
```

```
## 'data.frame':    1565652 obs. of  9 variables:
##  $ Barcode   : chr  "LJE04" "LJE04" "LJE04" "LJE04" ...
##  $ Well      : chr  "B8f1" "B8f1" "B8f1" "B8f1" ...
##  $ SampleID  : chr  "KW3_1" "KW3_1" "KW3_1" "KW3_1" ...
##  $ FeatureSet: chr  "B" "B" "B" "B" ...
##  $ TargetName: chr  "hsa-miR-600_001556" "hsa-miR-600_001556" "hsa-miR-600_001556" "hsa-miR-600_001556" ...
##  $ Cycle     : int  1 2 3 4 5 6 7 8 9 10 ...
##  $ Rn        : num  913 920 916 927 920 ...
##  $ dRn       : num  11.1 16.9 15.4 23 19.9 ...
##  $ NumCycle  : num  40 40 40 40 40 40 40 40 40 40 ...
```

A new method should take these data as input and produce an expression estimate and quality metric for each feature and each sample. These should be stored in a list with two elements: `ct` containing the expression estimates and `qc` containing the quality metrics. The row and column names should match those in the example data sets (*lifetech* and *qpcRdefault*).

# 4 Session Info

```
sessionInfo()
```

```
## R version 3.2.2 (2015-08-14)
## Platform: x86_64-apple-darwin13.4.0 (64-bit)
## Running under: OS X 10.9.5 (Mavericks)
## 
## locale:
## [1] en_US.UTF-8/en_US.UTF-8/en_US.UTF-8/C/en_US.UTF-8/en_US.UTF-8
## 
## attached base packages:
## [1] parallel  stats     graphics  grDevices utils     datasets  methods  
## [8] base     
## 
## other attached packages:
## [1] miRcomp_1.1.6       miRcompData_1.0.0   Biobase_2.30.0     
## [4] BiocGenerics_0.16.1 BiocStyle_1.8.0    
## 
## loaded via a namespace (and not attached):
##  [1] digest_0.6.8       formatR_1.2.1      magrittr_1.5      
##  [4] evaluate_0.8       KernSmooth_2.23-15 stringi_1.0-1     
##  [7] rmarkdown_0.8.1    tools_3.2.2        stringr_1.0.0     
## [10] yaml_2.1.13        htmltools_0.2.6    knitr_1.11
```
