# Supplementary figures and images for: A benchmark for microRNA quantification algorithms using the OpenArray platform
Source: BMC Bioinformatics. 2016 Mar 22;17:138. doi: 10.1186/s12859-016-0987-8 (PMC4802579; doi:10.1186/s12859-016-0987-8)

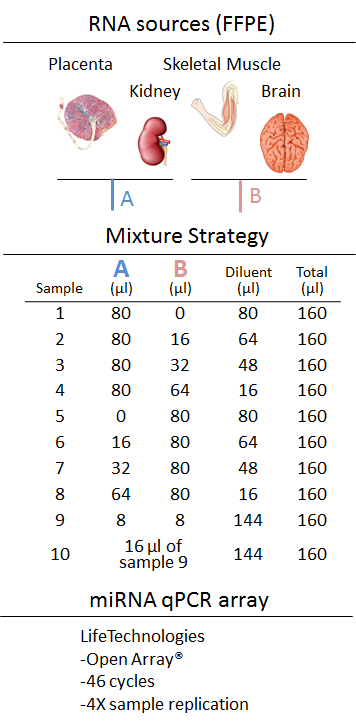

Supplement: Additional file 4 — miRcomp R package source. The source code for the miRcomp R package, also available at: http://bioconductor.org/packages/miRcomp/. (GZ 1802 kb) [file 12859_2016_987_MOESM4_ESM.gz › miRcomp/vignettes/design.png]
