# Supplementary material for: A benchmark for microRNA quantification algorithms using the OpenArray platform
Source: BMC Bioinformatics. 2016 Mar 22;17:138. doi: 10.1186/s12859-016-0987-8 (PMC4802579; doi:10.1186/s12859-016-0987-8)
Supplement: Additional file 6 — TaqMan OpenArray MicroRNA Panels User Guide. The technical manual of the source code for the TaqMan OpenArray MicroRNA Panels, also available at: https://tools.thermofisher.com/content/sfs/manuals/cms_092509.pdf. (PDF 996 KB) [file 12859_2016_987_MOESM6_ESM.pdf]

USER GUIDE

applied biosystems by life technologies™

TaqMan® OpenArray® MicroRNA Panels

OpenArray® Plates for microRNA expression analysis

for use with: Megaplex™ Primer Pools

TaqMan® OpenArray® Human MicroRNA Panel
TaqMan® OpenArray® Rodent MicroRNA Panel

Catalog Numbers 4461104, 4461105
Publication Part Number 4461306 Rev. C
Revision Date June 2011

life technologies™

For Research Use Only. Not intended for any animal or human therapeutic or diagnostic use.

Information in this document is subject to change without notice.

APPLIED BIOSYSTEMS DISCLAIMS ALL WARRANTIES WITH RESPECT TO THIS DOCUMENT, EXPRESSED OR IMPLIED, INCLUDING BUT NOT LIMITED TO THOSE OF MERCHANTABILITY OR FITNESS FOR A PARTICULAR PURPOSE. TO THE FULLEST EXTENT ALLOWED BY LAW, IN NO EVENT SHALL APPLIED BIOSYSTEMS BE LIABLE, WHETHER IN CONTRACT, TORT, WARRANTY, OR UNDER ANY STATUTE OR ON ANY OTHER BASIS FOR SPECIAL, INCIDENTAL, INDIRECT, PUNITIVE, MULTIPLE OR CONSEQUENTIAL DAMAGES IN CONNECTION WITH OR ARISING FROM THIS DOCUMENT, INCLUDING BUT NOT LIMITED TO THE USE THEREOF, WHETHER OR NOT FORESEEABLE AND WHETHER OR NOT APPLIED BIOSYSTEMS IS ADVISED OF THE POSSIBILITY OF SUCH DAMAGES.

Research Use Only

The purchase of this product conveys to the purchaser the limited, non-transferable right to use the purchased amount of the product only to perform internal research for the sole benefit of the purchaser. No right to resell this product or any of its components is conveyed expressly, by implication, or by estoppel. This product is for internal research purposes only and is not for use in commercial applications of any kind, including, without limitation, quality control and commercial services such as reporting the results of purchaser's activities for a fee or other form of consideration. For information on obtaining additional rights, please contact outlicensing@lifetech.com or Out Licensing, Life Technologies, 5791 Van Allen Way, Carlsbad, California 92008.

© 2011 Life Technologies Corporation. All rights reserved. The trademarks mentioned herein are the property of Life Technologies Corporation or their respective owners. TaqMan is a registered trademark of Roche Molecular Systems, Inc. Integromics and RealTime StatMiner are registered trademarks of Integromics, S.L.

Part Number 4461306 Rev C

June 2011

Contents

About this Guide 5

TaqMan® OpenArray® MicroRNA Panels 7

Product information 7
Required materials and equipment 7
Workflow 9
Before you begin 10
Reverse transcribe the RNA with Megaplex™ RT Primers 13
Preamplify the cDNA with Megaplex™ PreAmp Primers 14
Run the TaqMan® OpenArray® MicroRNA Panels 16
Analyze the data 18
Troubleshooting 18

APPENDIX A Good Laboratory Practices 19

Working with RNA 19
Preparing samples for PCR amplification 19

APPENDIX B Safety 21

Chemical safety 21
Biological hazard safety 22

Documentation and Support 23

Related documentation 23
Obtaining SDSs 23
Obtaining support 24

About this Guide

This guide provides instructions for:

- Reverse transcription (RT) of microRNA-containing total RNA with Megaplex™ RT Primers,
- Preamplification of the RT products with Megaplex™ PreAmp Primers, and
- Real-time PCR analysis using TaqMan® OpenArray® MicroRNA Panels.

These procedures enable microRNA expression profiling of a biological sample of interest.

The user should be familiar with laboratory practices for handling RNA and have a working knowledge of real-time PCR laboratory techniques, instrumentation, and software.

IMPORTANT! Before using this product, read and understand the information in the "Safety" appendix in this document.

TaqMan® OpenArray® MicroRNA Panels

Product information

Purpose

TaqMan® OpenArray® MicroRNA Panels enable simultaneous running of hundreds of TaqMan® MicroRNA Assays for human (human panel) or mouse and rat (rodent panel) species in a plate format on the Applied Biosystems OpenArray® Real-Time PCR System. These panels require the use of matching Megaplex™ Primer Pools (composed of Megaplex™ RT Primers and Megaplex™ PreAmp Primers) for microRNA (miRNA) reverse transcription and preamplification prior to real-time PCR using the TaqMan® OpenArray® MicroRNA Panel.

First, mature miRNAs present in an RNA sample are reverse transcribed into cDNA using **Megaplex™ RT Primers** in a set of two predefined pools (Pool A and Pool B). Each pool contains up to 381 stem-looped reverse-transcription (RT) primers.

Next, the cDNA products undergo unbiased PCR preamplification using **Megaplex™ PreAmp Primers** in a set of two pools (Pool A and Pool B) of gene-specific forward and reverse primers.

The preamplified cDNA products then undergo real-time PCR amplification and analysis using **TaqMan® OpenArray® MicroRNA Panels** in 3072-well microfluidic OpenArray® Plates containing dried TaqMan® primers and probes for up to 758 miRNAs and controls.

This workflow is presented on page 9.

Contents and storage

Each panel accommodates three RNA samples.

TaqMan® OpenArray® MicroRNA Panel	Part no.	Contents	Storage
TaqMan® OpenArray® Human MicroRNA Panel	4461104	One panel	Store at –15 to –25°C and protect from light.
TaqMan® OpenArray® Rodent MicroRNA Panel	4461105		

Download the OpenArray® Plate setup file (in .tpf format) for the specific TaqMan® OpenArray® MicroRNA Panel from the OpenArray® Plate product page at **www.lifetechnologies.com** (Applied Biosystems).

Required materials and equipment

Unless otherwise noted, all materials and equipment are available at **www.lifetechnologies.com** (Applied Biosystems).

Megaplex™ Primer Pools

Megaplex™ Primer Pool Sets include content-matched pools of Megaplex™ RT Primers and Megaplex™ PreAmp Primers.

Store Megaplex™ Primer Pools at –15 to –25°C.

For this TaqMan® OpenArray® MicroRNA Panel...	...Use these Megaplex™ Primer Pools	
	Product	Contents
TaqMan® OpenArray® Human MicroRNA Panel	Megaplex™ Primer Pools, Human Pools Set v3.0 (4444750)	• Megaplex™ RT Primers Pool A† • Megaplex™ RT Primers Pool B† • Megaplex™ PreAmp Primers Pool A‡ • Megaplex™ PreAmp Primers Pool B‡
TaqMan® OpenArray® Rodent MicroRNA Panel	Megaplex™ Primer Pools, Rodent Pools Set v3.0 (4444766)	

† 50 µL of stem-loop RT primers, 1.5 mL 25 mM $MgCl_2$.
‡ 150 µL of forward and reverse primers

Other required materials

Item	Part no.
TaqMan® MicroRNA Reverse Transcription Kit	• 4366596 (200 rxns) • 4366597 (1000 rxns)
TaqMan® PreAmp Master Mix	4391128
TaqMan® OpenArray® Real-Time PCR Master Mix	• 4462159 (1.5 mL) • 4462164 (5 ml)
OpenArray® Real-Time PCR Accessories Kit	4453975
MicroAmp® Optical 96-Well Reaction Plate	4316813
OpenArray® 384-Well Sample Plates	• 4406947 • 4453929 (Barcoded)
MicroAmp® Clear Adhesive Film	4306311
Costar Aluminum Sealing Tape, for sealing the OpenArray® 384-Well Sample Plates	Corning Life Sciences, 07200683
0.1✕ TE pH 8.0	User-supplied

Required equipment

- General lab equipment: vortexer, pipettors and tips, etc. (obtained through major laboratory suppliers)
- The OpenArray® AccuFill™ System and accessories, required for loading the TaqMan® OpenArray® MicroRNA Panels

Item	Part no.
OpenArray® AccuFill™ System	4457243
OpenArray® AccuFill™ System Tips	• 4457246 (1 pack) • 4458107 (10 pack)
OpenArray® Real-Time PCR Plate Frame.	4453942 (3-pack)

- Thermal cycler, required for the RT and preamplification reactions
- OpenArray® Real-Time PCR System, required for running the TaqMan® OpenArray® MicroRNA Panels

Optional materials

The *mir*Vana™ miRNA Isolation Kit (Part no. AM1560) is recommended for preparing high-quality total RNA containing miRNA species. Follow the total RNA isolation procedure (do *not* follow the option for enrichment for small RNAs).

Workflow

For a full miRNA profile, run two Megaplex™ RT reactions (Pools A and B) and two corresponding preamplification reactions (Pool A and B) per RNA sample (two *sample-pools*). A full miRNA profile takes one-third of an TaqMan® OpenArray® MicroRNA Panel; a TaqMan® OpenArray® MicroRNA Panel can accommodate up to three individual samples, or a sample with three replicates.

Reverse transcribe the RNA with Megaplex™ RT Primers (page 13)

Set up the RT reactions

↓

Run the RT reactions

↓

(*Optional*) Stopping point

↓

Preamplify the cDNA with Megaplex™ PreAmp Primers (page 14)

Set up the preamplification reactions

↓

Run the preamplification reaction

↓

Dilute the preamplification products

↓

(*Optional*) Stopping point

↓

Run the TaqMan® OpenArray® MicroRNA Panels (page 16)

Access the setup files and prepare the TaqMan® OpenArray® MicroRNA Panel

↓

Prepare the PCR Reaction Mixes for each sample

↓

Load and run the TaqMan® OpenArray® MicroRNA Panel

↓

Analyze the data (page 18)

Before you begin

Plate overview

This procedure requires three types of plates:

- MicroAmp® Optical 96-Well Reaction Plate (*96-well plate*)
- OpenArray® 384-Well Sample Plate (*384-well plate*)
- TaqMan® OpenArray® MicroRNA Panel (*OpenArray® Plate*)

MicroAmp® Optical 96-Well Reaction Plate

This is a 96-well thermocycling reaction plate. 96-well plates are used in the following procedures:

- Megaplex™ RT reactions (page 14)
- Preamplification with Megaplex™ PreAmp Primers (two plates) (page 14)
- Dilution of the preamplification products (page 16)
- Preparation of the PCR Reaction Mix with each preamplification product (page 16)

OpenArray® 384-Well Sample Plate

384-well plates are used when setting up the real-time PCR reactions as an intermediate plate prior to loading each PCR Reaction Mix into the TaqMan® OpenArray® MicroRNA Panel using the OpenArray® AccuFill™ System (page 17).

Figure 1 OpenArray® 384-well Sample Plate layout

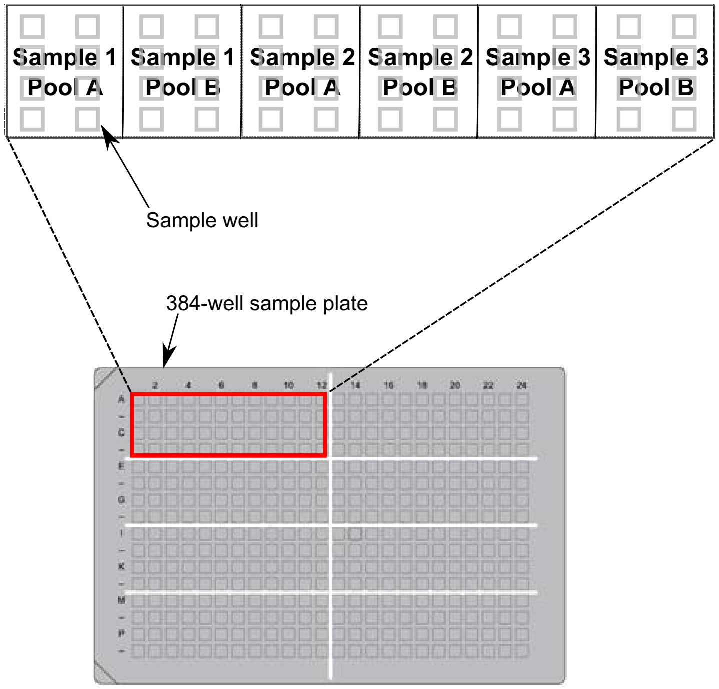

As shown in Figure 1 on page 10, the OpenArray® 384-Well Sample Plate can be divided into eight regions, each containing 48 wells. Each 48-well region is used to fill one TaqMan® OpenArray® MicroRNA Panel. Each well on the 384-well plate is used to load a subarray of 64 through-holes on the OpenArray® Plate (see Figure 2).

IMPORTANT! The well dimensions of the OpenArray® 384-Well Sample Plates are specifically suited for use with the AccuFill™ System. We do not recommend the use of other microtiter plates.

TaqMan® OpenArray® MicroRNA Panel

The TaqMan® OpenArray® MicroRNA Panels is an OpenArray® Plate. As shown in Figure 2, an OpenArray® Plate is divided into 48 subarrays; each subarray consists of 64 through-holes. An entire sub-array (64 through-holes) is loaded from each well in the OpenArray® 384-Well Sample Plate using the AccuFill™ System.

Through-holes are preloaded with TaqMan® primers and probes for human or rodent microRNAs, or for endogenous control RNAs. Individual through-holes accommodate 33-nL reaction volumes; hydrophilic and hydrophobic coatings enable reagents to be held within the through-holes.

Figure 2 TaqMan® OpenArray® MicroRNA Panel layout

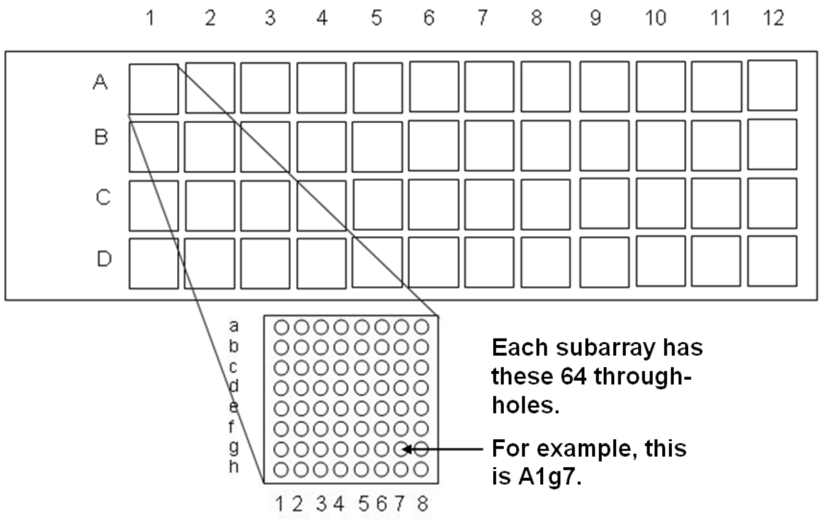

Plate loading scheme

The plates are loaded according to the capacity of a single TaqMan® OpenArray® MicroRNA Panel, as illustrated in Figure 3 on page 12. Each TaqMan® OpenArray® MicroRNA Panel can accommodate three RNA samples (or one sample in triplicate) for a full miRNA profile using Megaplex™ Primer Pools A and B.

We recommend using 96-well plates for the RT and preamplification reactions, and OpenArray® 384-Well Sample Plates to prepare the PCR Reaction Mix for loading into the TaqMan® OpenArray® MicroRNA Panel.

A single 96-well plate can hold up to 48 samples (each run against Pool A and Pool B). These 48 samples would be sufficient to fill two 384-well plates, which in turn will load a total of 16 TaqMan® OpenArray® MicroRNA Panels.

Figure 3 Plate loading scheme

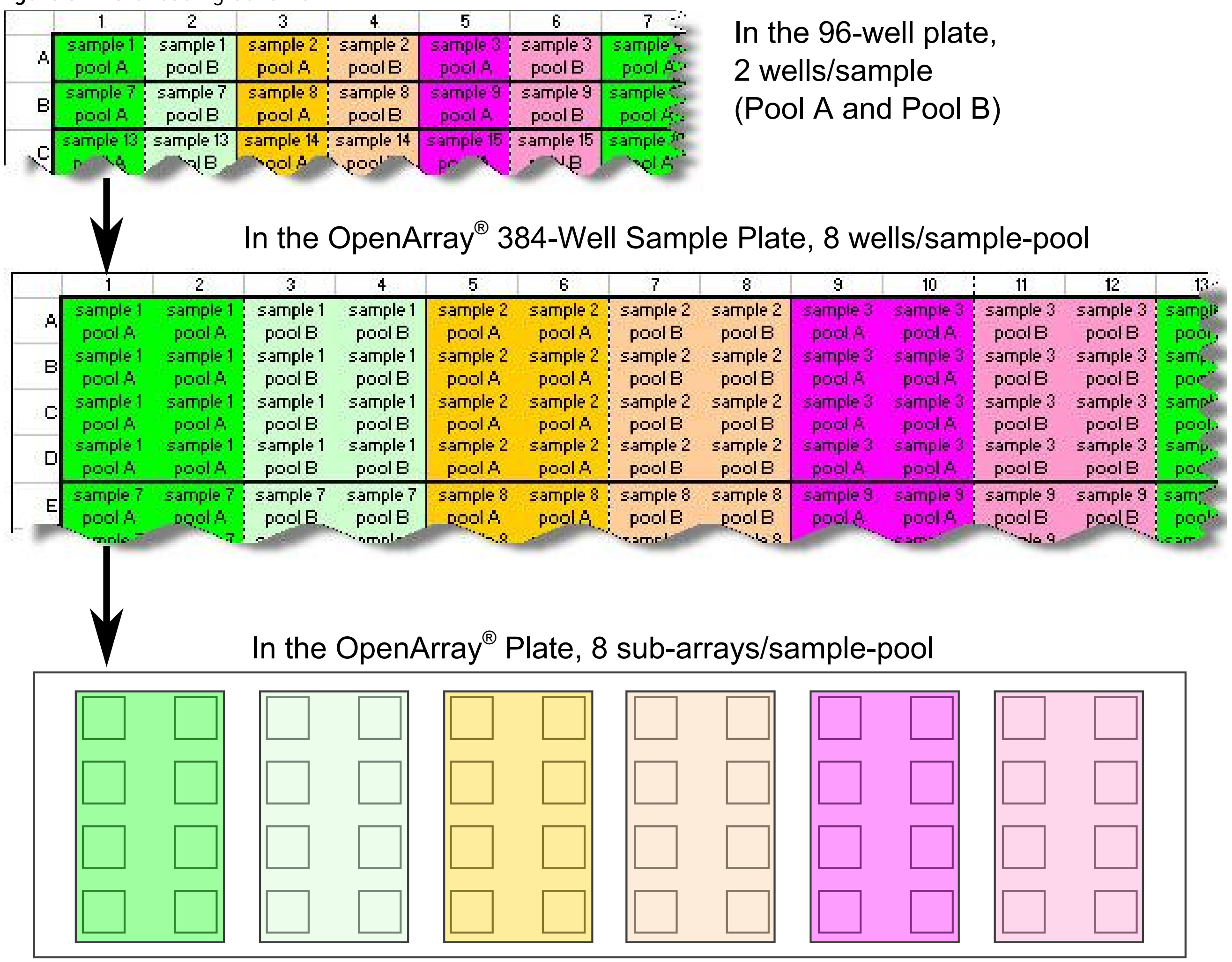

Input RNA requirements

Start with high-quality total RNA containing small RNA. Isolate RNA using kits or reagents appropriate for your application. We recommend the *mir*Vana™ miRNA Isolation Kit (Part no. AM1560), using the total RNA isolation procedure.

IMPORTANT! The method used to isolate the total RNA must preserve the small RNA fraction. However, in order to preserve endogenous control sequences in the total RNA, do *not* enrich for the small RNA fraction. This may cause longer control transcripts (snoRNAs) to be lost. For example, if you use the *mir*Vana™ miRNA Isolation Kit, follow the total RNA isolation procedure, not the small RNA enrichment procedure.

For each Megaplex™ RT Primers pool, the RT reaction with downstream preamplification supports 50–200 ng of input total RNA.

For most tissues, 100 ng of total RNA per Megaplex™ RT Primers pool produces a comprehensive miRNA profile. We recommend verifying the optimal input quantity of total RNA for your sample type.

Reverse transcribe the RNA with Megaplex™ RT Primers

About the RT reactions

In this step, single-stranded cDNA is reverse transcribed from total RNA. Run two reverse transcription (RT) reactions per sample, using Megaplex™ RT Primers Pools A and B.

Each reverse transcription (RT) reaction has a final volume of 7.5 µL and contains:

- 100 ng (recommended) of total RNA in 3 µL
- 4.5 µL of RT Reaction Mix, containing reverse transcriptase, Megaplex™ RT Primers Pool A or Pool B, and other reverse transcription reagents

Set up the RT reactions

1. Thaw the following on ice:
   - Megaplex™ RT Primers
   - TaqMan® MicroRNA Reverse Transcription Kit components (do not vortex the MultiScribe™ Reverse Transcriptase)
   - $MgCl_2$ (supplied with the Megaplex™ RT Primers)
2. Combine the following in each of two 1.5-mL microcentrifuge tubes (one for Pool A, the other for Pool B):

RT Reaction Mix Components	Volume per reaction	Volume for 3 reactions‡
Megaplex™ RT Primers (10×), Pool A or Pool B†	0.75 µL	2.5 µL
dNTPs with dTTP (100 mM)	0.15 µL	0.5 µL
MultiScribe™ Reverse Transcriptase (50 U/µL)	1.50 µL	5.1 µL
10× RT Buffer	0.75 µL	2.5 µL
$MgCl_2$ (25 mM)	0.90 µL	3.0 µL
RNase Inhibitor (20 U/µL)	0.09 µL	0.3 µL
Nuclease-free water	0.35 µL	1.2 µL
Total	4.50 µL	15.1 µL

† Use Pool A in one tube, and Pool B in the other.
‡ Includes 12.5% excess for loss from pipetting.

3. Pipet up and down to mix, then centrifuge the tubes briefly.
4. Transfer 4.5 µL of the RT Reaction Mix into the appropriate number of wells of a 96-well MicroAmp® Optical Reaction Plate.

   Note: Each RNA sample is processed in two wells: one for Pool A and one for Pool B. Thus each 96-well plate can process 48 samples. See Figure 3, "Plate loading scheme" on page 12.
5. Add 100 ng of total RNA (recommended amount) in 3 µL of solution to each well containing RT Reaction Mix.

   Note: You can use 3 µL of water for the No Template Control reactions.

6. Depending on the number of RT reactions, mix the reactions in one of these ways:
   - Pipet each mixture up and down a few times, then seal the plate using MicroAmp® Clear Adhesive Film.
   - Seal the plate using MicroAmp® Clear Adhesive Film, then invert the plate 6 times.

   Note: Do not use MicroAmp® *Optical* Adhesive Film to seal the plate.
7. Spin the plate briefly to collect the contents at the bottom of the wells, then incubate the plate on ice for 5 minutes.

Run the RT reactions

1. Set up the run method in a thermal cycler using the following conditions:
   - Ramp speed or mode: **9700** using **Std** or **Max** ramp speed.
   - Reaction volume (µL): **7.5** (enter 8 µL if your instrument accepts only whole number values)
   - Thermal cycling conditions:

Stage	Temp	Time
Cycle (40 Cycles)	16°C	2 min
	42°C	1 min
	50°C	1 sec
Hold	85°C	5 min
Hold	4°C	∞

2. Load, then run the plate.

STOPPING POINT (Optional) The RT product (cDNA) can be stored at -15 to -25°C for at least one week.

Preamplify the cDNA with Megaplex™ PreAmp Primers

About the preamplification reaction

In this step, specific cDNA targets are preamplified to increase the quantity of desired cDNA prior to PCR on the TaqMan® OpenArray® MicroRNA Panels.

Each preamplification reaction has a final volume of 25 µL and contains:

- 2.5 µL RT product (cDNA) from "Run the RT reactions"
- 22.5 µL PreAmp Reaction Mix, containing Megaplex™ PreAmp Primers Pool A or Pool B and TaqMan® PreAmp Master Mix

  Use Megaplex™ PreAmp Primers Pools A or B corresponding to the Megaplex™ RT Primers Pool used for reverse transcription.

Set up the preamplification reactions

1. Thaw the Megaplex™ PreAmp Primers on ice and mix by inverting 6 times. Spin briefly to collect the contents at the bottom of the tubes.
2. Mix the TaqMan® PreAmp Master Mix (2X) by swirling the bottle.

3. Prepare PreAmp Reaction Mix, one for Pool A and one for Pool B, by combining the following in each of two 1.5-mL microcentrifuge tubes:

PreAmp Reaction Mix components	Volume per reaction	Volume for 3 reactions†
TaqMan® PreAmp Master Mix (2X)	12.5 µL	42.4 µL
Megaplex™ PreAmp Primers (10X), Pool A or Pool B‡	2.5 µL	8.4 µL
Nuclease-free water	7.5 µL	25.3 µL
Total	22.5 µL	76.1 µL

† Includes 12.5% excess for volume loss from pipetting.
‡ Use Pool A in one tube, and Pool B in the other.

4. Pipet up and down to mix, then centrifuge the tubes briefly.
5. Pipet 2.5 µL of each RT product into a well of a MicroAmp® Optical 96-well Reaction Plate. (Two wells per RNA sample, one for the Pool A RT product and the other for the Pool B product.)
6. Dispense 22.5 µL of PreAmp Reaction Mix into each well of the 96-well plate containing the corresponding RT product (pool A or pool B).
7. Depending on the number of preamplification reactions, mix the reactions in one of these ways:
   - Pipet each mixture up and down a few times, then seal the plate using MicroAmp® Clear Adhesive Film.
   - Seal the plate using MicroAmp® Clear Adhesive Film, then invert the plate 6 times.
8. Spin the plate briefly to collect the contents at the bottom of the wells, then incubate the plate on ice for 5 minutes.

Run the preamplification reaction

Set up the run method using the following conditions:

- Ramp speed or mode: **9700** using **Std** ramp speed.
- Reaction volume (µL): **25**
- Thermal-cycling parameters:

Stage	Temp	Time
Hold	95°C	10 min
Hold	55°C	2 min
Hold	72°C	2 min
Cycle (12 Cycles)	95°C	15 sec
	60°C	4 min
Hold†	99.9°C	10 min
Hold	4°C	∞

† Required for enzyme inactivation.

Dilute the preamplification products

1. Remove the 96-well plate from the thermal cycler and briefly centrifuge the plate.
2. For each preamplification reaction, add 156 µL of 0.1× TE pH 8.0 to one well of a new 96-well plate.
3. Transfer 4 µL of each preamplification reaction to a well containing 0.1× TE buffer (final dilution: 1 to 40).
4. Depending on the number of preamplification reactions, mix the diluted products in one of these ways:
   - Pipet up and down a few times, then seal the plate using MicroAmp® Clear Adhesive Film.
   - Seal the plate using MicroAmp® Clear Adhesive Film, then invert the plate 6 times.
5. Spin the plate briefly to collect the contents at the bottom of the wells, then place the plate on ice.

   Note: The diluted preamplification product is stable for up to 12 hours at 4°C.

STOPPING POINT (Optional) Store the preamplified product (diluted or undiluted) at -15 to -25°C for up to one week.

Run the TaqMan® OpenArray® MicroRNA Panels

In this step, the preamplification products undergo real-time PCR in the TaqMan® OpenArray® MicroRNA Panel.

Access the setup files and prepare the TaqMan® OpenArray® MicroRNA Panel

1. Download the OpenArray® Plate setup file (in .tpf format) for the TaqMan® OpenArray® MicroRNA Panel from the OpenArray® Plate product page at **www.lifetechnologies.com** (Applied Biosystems).

   The serial number (S/N) from one TaqMan® OpenArray® MicroRNA Panel per order is required to download the correct files.
2. Remove the TaqMan® OpenArray® MicroRNA Panel from the freezer and allow it to come to room temperature. This should take approximately 15 minutes.

Prepare the PCR Reaction Mixes for each sample

For each sample, use the diluted preamplification product A and B and TaqMan® OpenArray® Real-Time PCR Master Mix to prepare PCR Reaction Mix A and B.

Note: We recommend storing TaqMan® OpenArray® Real-Time PCR Master Mix at –15°C to –25°C until first use, then store at 4°C. If frozen, fully thaw on ice prior to use.

Note: All volumes in this section are calculated to include 12.5% excess volume to accommodate loss from pipetting.

1. If the diluted preamplification products were stored frozen, thaw the plate completely on ice. Mix by inverting the sealed plate 6 times or by vortexing gently, then centrifuge the plate briefly.
2. Mix the TaqMan® OpenArray® Real-Time PCR Master Mix by swirling the bottle.

3. For each sample, pipet 22.5 µL of TaqMan® OpenArray® Real-Time PCR Master Mix into each of two adjacent wells (one for Pool A and one for Pool B) of a clean 96-well plate. See Figure 3 on page 12 for detailed layout recommendations.
4. For each sample, pipet 22.5 µL of diluted Pool A preamplification product into one well of each pair, and 22.5 µL of diluted Pool B preamplification product into the other well.
5. Seal the plate, vortex gently to mix, and centrifuge the plate briefly.

   Note: The assembled PCR Reaction Mix can be kept at 4°C for up to 12 hours in the 96-well plate before transferring to the 384-well plate, described in the next section.

Load and run the TaqMan® OpenArray® MicroRNA Panel

For detailed information about loading and sealing the TaqMan® OpenArray® MicroRNA Panel using the OpenArray® AccuFill™ System, refer to the *OpenArray® AccuFill™ System User Guide* (Part no. 4456986). For information on running PCR with TaqMan® OpenArray® MicroRNA Panels, refer to the *OpenArray® Real-Time PCR System User Guide* (Part no. 4458837).

1. Dispense 5 µL of each PCR Reaction Mix into each of 8 wells on an OpenArray® 384-Well Sample Plate, as shown on the plate map below (each square represents eight sample wells). Use a second 384-well sample plate if needed to hold all samples. Refer to Figure 3 on page 12.

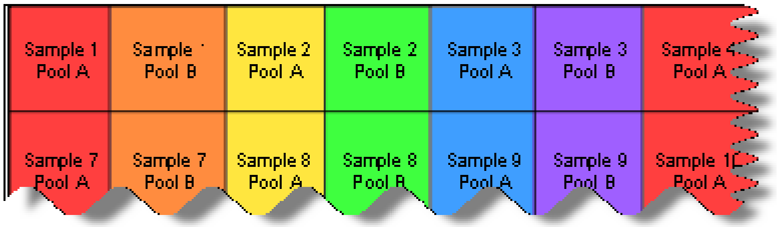

2. After the 384-well plate is filled with the PCR Reaction Mixes, and the TaqMan® OpenArray® MicroRNA Panel has reached room temperature, carefully remove the panel from its packaging.
3. Load the TaqMan® OpenArray® MicroRNA Panel from the 384-well plate, using the standard AccuFill™ method.
4. Run the TaqMan® OpenArray® MicroRNA Panels using the downloaded .tpf file corresponding to the serial number for each plate.

Note: TaqMan® OpenArray® MicroRNA Panels must be run within one hour of loading.

Analyze the data

For detailed information on how to analyze and export primary real-time PCR data, refer to the *OpenArray® Real-Time PCR System User Guide* (Part no. 4458837).

Export the data from the OpenArray® Real-Time qPCR Analysis Software following procedures described in the *OpenArray® Real-Time PCR System User Guide*. Export summary results: Select **File > Export Cycling Data**. Select the **Summary Results** checkbox in the dialog box.

Perform the analysis with DataAssist™ Software. DataAssist™ Software is a free download from the Applied Biosystems website. Search for **dataassist** at **www.appliedbiosystems.com**.

Troubleshooting

Observation	Possible Cause	Recommended Action
The C_T value for the No Template Control (NTC) is < 25 for some assays	Non-specific interactions between primers	For NTC information on a specific assay, refer to the Megaplex™ Assay Performance File.
	Contamination	Work in an amplicon-free environment.
	Improper dilution of the preamplification reaction prior to real-time PCR	Follow the recommendations in the protocol. See "Dilute the preamplification products" on page 16.
The C_T values for the endogenous controls are highly variable across the sample set	Natural biological variation	Use an alternative endogenous control such as a non-variable microRNA.<br>Use an alternative normalization strategy.
No amplification or poor amplification of samples	Low-abundance gene	Use more starting material.
	Use of non-Applied Biosystems reagents	Use Applied Biosystems reagents.
	Omission of reaction components, or deviation from the recommended protocol	• Add all required reaction components.<br>• Follow precise recommendations in protocol.
	Sample omitted from RT reaction	Add sample to RT reaction.
Poor reproducibility across technical replicates	Inadequate mixing of reagents	Make sure that all samples and reactions are mixed well by pipetting or mixing as described in the procedure.

A Good Laboratory Practices

Working with RNA

Before working with RNA:

- Clean the lab bench and pipettors with an RNase decontamination solution (for example, Ambion® RNase*Zap*® Solution, Part no. AM9780).
- Wear laboratory gloves for this procedure.
- Use RNase-free pipette tips to handle kit reagents, and avoid putting used tips into the reagent containers.

For more information about good laboratory practices for working with RNA, refer to Technical Bulletin #159, *Working with RNA*, at **www.invitrogen.com**.

Preparing samples for PCR amplification

- Use a positive-displacement pipette or aerosol-resistant pipette tips.
- Follow proper pipette-dispensing techniques to prevent aerosols.
- Wear clean gloves and a clean lab coat (not previously worn while handling amplified PCR products or used during sample preparation).
- Change gloves whenever you suspect that they are contaminated.
- Maintain separate areas and dedicated equipment and supplies for:
  - Sample preparation
  - PCR setup
  - PCR amplification
  - Analysis of PCR products
- Never bring amplified PCR products into the PCR setup area.
- Open and close all sample tubes carefully. Centrifuge tubes before opening. Try not to splash or spray PCR samples.
- Keep reactions and components capped as much as possible.
- Clean lab benches and equipment periodically with 10% bleach solution. Use DNA*Zap*™ Solution (Part no. AM9890).

B

Safety

Chemical safety

WARNING! GENERAL CHEMICAL HANDLING. To minimize hazards, ensure laboratory personnel read and practice the general safety guidelines for chemical usage, storage, and waste provided below, and consult the relevant SDS for specific precautions and instructions:

- Read and understand the Safety Data Sheets (SDSs) provided by the chemical manufacturer before you store, handle, or work with any chemicals or hazardous materials. To obtain SDSs, see the "Documentation and Support" section in this document.
- Minimize contact with chemicals. Wear appropriate personal protective equipment when handling chemicals (for example, safety glasses, gloves, or protective clothing).
- Minimize the inhalation of chemicals. Do not leave chemical containers open. Use only with adequate ventilation (for example, fume hood).
- Check regularly for chemical leaks or spills. If a leak or spill occurs, follow the manufacturer's cleanup procedures as recommended in the SDS.
- Ensure that the waste is stored, transferred, transported, and disposed of according to all local, state/provincial, and/or national regulations.

Biological hazard safety

WARNING! BIOHAZARD. Biological samples such as tissues, body fluids, infectious agents, and blood of humans and other animals have the potential to transmit infectious diseases. Follow all applicable local, state/provincial, and/or national regulations. Wear appropriate protective equipment, which includes but is not limited to: protective eyewear, face shield, clothing/lab coat, and gloves. All work should be conducted in properly equipped facilities using the appropriate safety equipment (for example, physical containment devices). Individuals should be trained according to applicable regulatory and company/institution requirements before working with potentially infectious materials. Read and follow the applicable guidelines and/or regulatory requirements in the following:

In the U.S.:

- U.S. Department of Health and Human Services guidelines published in Biosafety in Microbiological and Biomedical Laboratories found at: **www.cdc.gov/biosafety**
- Occupational Safety and Health Standards, Bloodborne Pathogens (29 CFR§1910.1030), found at: **www.access.gpo.gov/nara/cfr/waisidx_01/29cfr1910a_01.html**
- Your company's/institution's Biosafety Program protocols for working with/handling potentially infectious materials.
- Additional information about biohazard guidelines is available at: **www.cdc.gov**

In the EU:

Check local guidelines and legislation on biohazard and biosafety precaution and refer to the best practices published in the World Health Organization (WHO) Laboratory Biosafety Manual, third edition, found at: **www.who.int/csr/resources/publications/biosafety/WHO_CDS_CSR_LYO_2004_11/en/**

Documentation and Support

Related documentation

The following related documents are shipped with TaqMan® OpenArray® MicroRNA Panels.

Document	Description	Part no.
TaqMan® OpenArray® MicroRNA Panels Quick Reference	Provides brief, step-by-step instructions for reverse transcription, preamplification, and real-time PCR using TaqMan® OpenArray® MicroRNA Panels.	4461310
OpenArray® Plates Product Insert	Provides inventory and storage information for OpenArray® Plates.	4461305

The following documents provide information about other components of the OpenArray® Real-Time PCR System.

Document	Description	Part no.
TaqMan® OpenArray® Real-Time PCR Plates User Guide	Provides procedures for preparing the TaqMan® OpenArray® Real-Time PCR Plates.	4458840
OpenArray® AccuFill™ System User Guide	Provides procedures for using the OpenArray® AccuFill™ System to load OpenArray® Plates.	4456986
OpenArray® Real-Time PCR System User Guide	Provides procedures for imaging and analyzing OpenArray® Plates. Provides maintenance information for the OpenArray® Real-Time PCR System.	4458837
OpenArray® Real-Time PCR System Quick Reference: Workflow	Describes the overall workflow and provides brief procedures for performing gene expression experiments with the OpenArray® Real-Time PCR System.	4458838

Note: For additional documentation, see "Obtaining support" on page 24.

Obtaining SDSs

Safety Data Sheets (SDSs) are available from **www.lifetechnologies.com/sds**.

Note: For the SDSs of chemicals not distributed by Life Technologies, contact the chemical manufacturer.

Obtaining support

For the latest services and support information for all locations, go to:

www.lifetechnologies.com/support

At the website, you can:

- Access worldwide telephone and fax numbers to contact Technical Support and Sales facilities
- Search through frequently asked questions (FAQs)
- Submit a question directly to Technical Support
- Search for user documents, SDSs, vector maps and sequences, application notes, formulations, handbooks, certificates of analysis, citations, and other product support documents
- Obtain information about customer training
- Download software updates and patches

Headquarters
5791 Van Allen Way | Carlsbad, CA 92008 USA | Phone +1 760 603 7200 | Toll Free in USA 800 955 6288
For support visit www.appliedbiosystems.com/support

www.lifetechnologies.com

life technologies™
