# Supplementary material for: A benchmark for microRNA quantification algorithms using the OpenArray platform
Source: BMC Bioinformatics. 2016 Mar 22;17:138. doi: 10.1186/s12859-016-0987-8 (PMC4802579; doi:10.1186/s12859-016-0987-8)
Supplement: Additional file 7 — Supplementary Figures. A PDF containing 7 Supplementary Figures referred to in this manuscript. (PDF 770 kb) [file 12859_2016_987_MOESM7_ESM.pdf]

Supplementary Figures

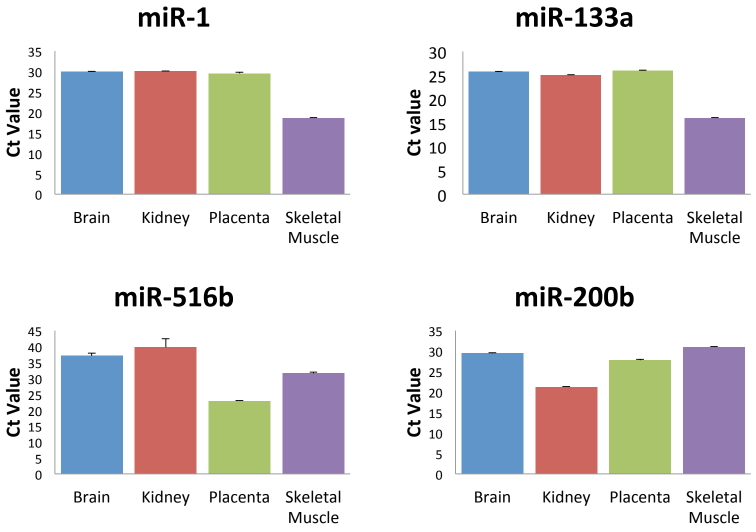

Figure 1: Confirmation of the presence of several tissue-enriched miRNAs by stem-loop reverse transcription quantitative PCR. As expected, miR-1 and miR-133a were enriched in skeletal muscle, miR-516b was enriched in placenta, and miR-200b was enriched in kidney.

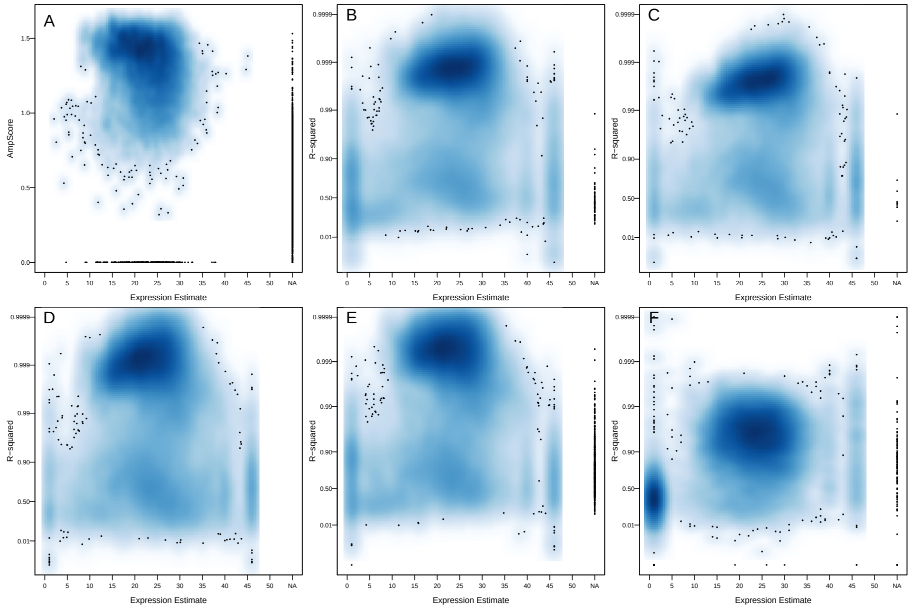

Figure 2: Similar to Figure 2 but expanded to show the relationship between quality and expression for six algorithms: (A) Life Technologies, (B) 4 parameter log sigmodial method, (C) 4 parameter sigmodial method, (D) 5 parameter sigmodial method, (E) 5 parameter log sigmodial method, (F) linear exponential method. For methods B-F, the R-squared values are plotted on the complementary log-log scale to improve visibility in the region of interest. Each point represents a single expression measurement and corresponding quality measure for a unique miRNA / sample combination. Two-dimensional scatterplot smoothing is used to avoid overplotting and convey the distribution of points across the plotting region.

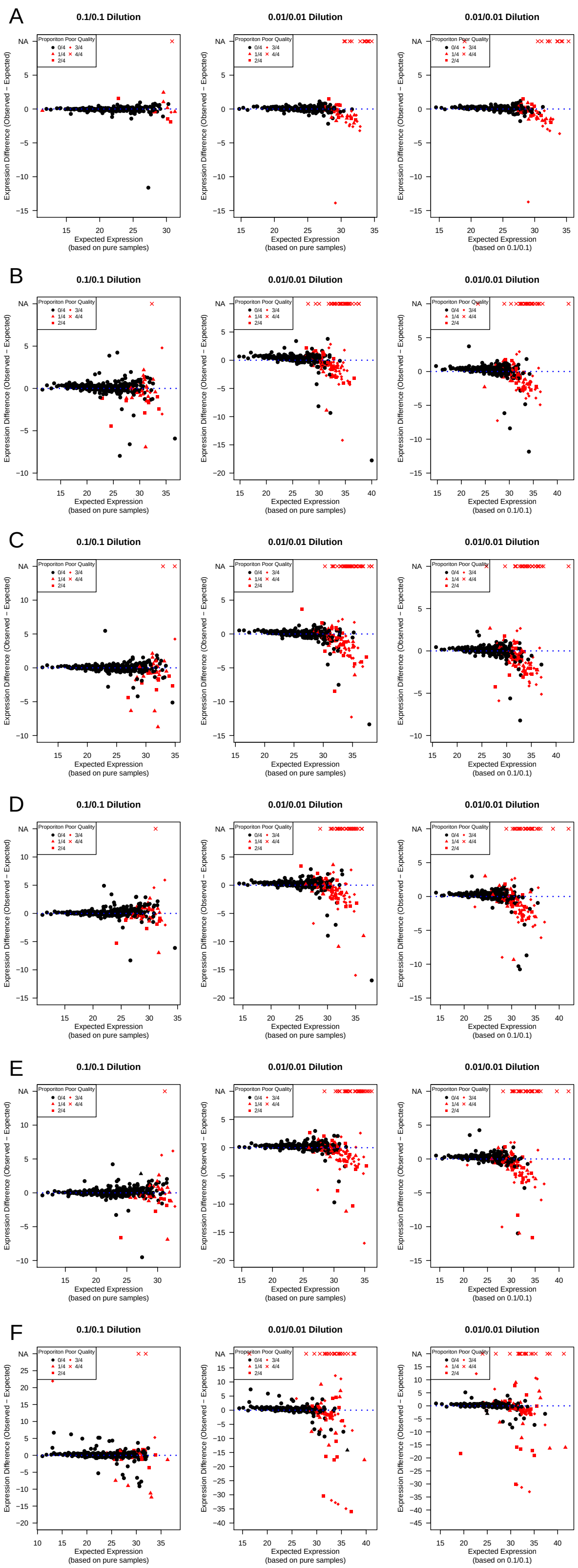

Figure 3: Similar to Figure 5 but expanded to show results from six algorithms: (A) Life Technologies, (B) 4 parameter log sigmodial method, (C) 4 parameter sigmodial method, (D) 5 parameter sigmodial method, (E) 5 parameter log sigmodial method, (F) linear exponential method.

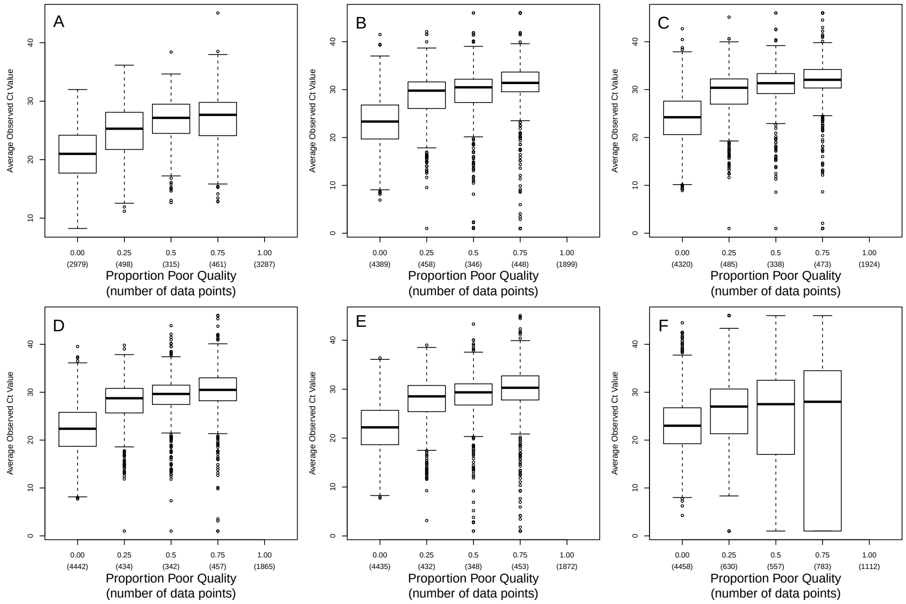

Figure 4: Similar to Figure 5 but expanded to show results from six algorithms: (A) Life Technologies, (B) 4 parameter log sigmodial method, (C) 4 parameter sigmodial method, (D) 5 parameter sigmodial method, (E) 5 parameter log sigmodial method, (F) linear exponential method. To further examine the limit of detection, we examine boxplots of average observed expression stratified by the proportion of poor quality data points. Below each box, we also report the number of unique feature / sample type combinations each box contains.

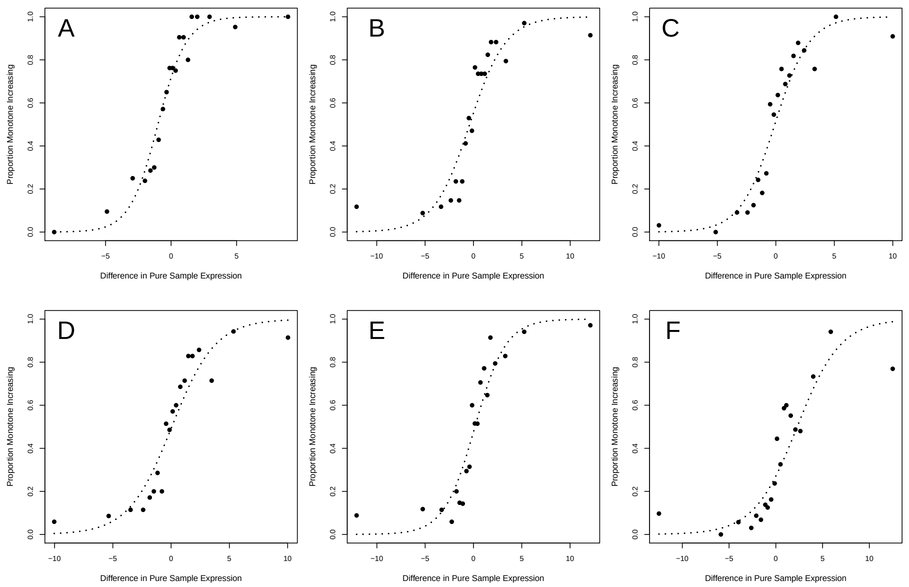

Figure 5: Similar to Figure 7 but expanded to show results from six algorithms: (A) Life Technologies, (B) 4 parameter log sigmodial method, (C) 4 parameter sigmodial method, (D) 5 parameter sigmodial method, (E) 5 parameter log sigmodial method, (F) linear exponential method. To examine the titration response, we plot the proportion of features that show monotone increasing expression as the amount of input RNA increases stratified by the difference in expression between the sample being titrated and the sample being held constant. Here we use samples 2-4 and 6-8 as two separate titration series. To assess the difference in expression between mixture components A and B, we use the expression estimates in the pure sample types: sample type 1 (pure A) and sample type 5 (pure B). Each point represents an average over the miRNAs falling within a given bin, where bins are defined based on the observed difference in pure sample expression (x-axis).

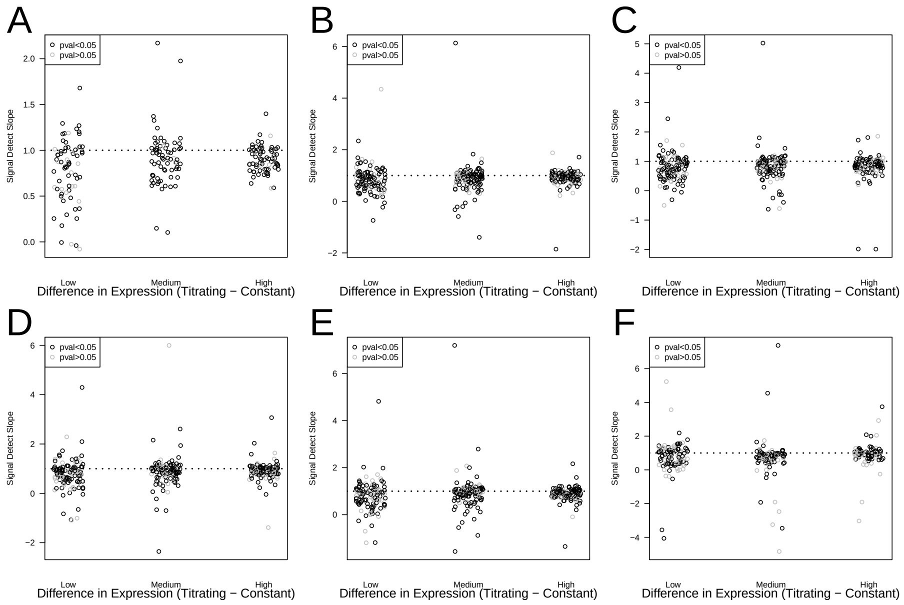

Figure 6: Similar to Figure 8 but expanded to show results from six algorithms: (A) Life Technologies, (B) 4 parameter log sigmodial method, (C) 4 parameter sigmodial method, (D) 5 parameter sigmodial method, (E) 5 parameter log sigmodial method, (F) linear exponential method. To assess accuracy, we calculate the signal detect slope: the slope of the regression line of observed expression on expected expression. The ideal signal detect slope is one, representing agreement between observed and expected expression. The signal detect slopes are stratified by pure sample expression. Each point represents a microRNA. Points in the figures are grey if the signal detect slope is not statistically signficantly different from zero. As such, a grey point corresponding to a signal detect slope well above zero represents a particularly noisy (large residual variance) response.

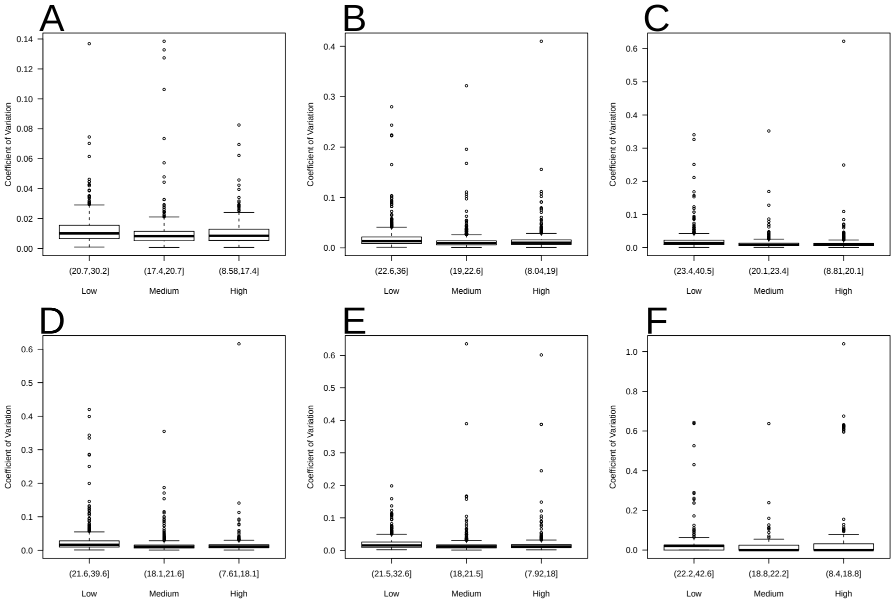

Figure 7: Similar to Figure 9 but expanded to show results from six algorithms: (A) Life Technologies, (B) 4 parameter log sigmodial method, (C) 4 parameter sigmodial method, (D) 5 parameter sigmodial method, (E) 5 parameter log sigmodial method, (F) linear exponential method. To assess precision, we calculate the within-replicate coefficient of variation, calculated as the within-replicate standard deviation divided by the within-replicate mean. These are calculated for each set of replicates (unique feature / sample type combination) that are of good quality and stratified by the observed expression.
